# Resonant Tender X‑ray Scattering for Disclosing the Backbone Conformation of Conjugated Polymers

**DOI:** 10.1021/acs.macromol.4c03107

**Published:** 2025-06-26

**Authors:** Yunfei Wang, Ka Hung Chan, Guillaume Freychet, Patryk Wąsik, Song Zhang, Zhiqiang Cao, Xiaodan Gu

**Affiliations:** † School of Polymer Science and Engineering, Center for Optoelectronic Materials and Devices, 5104 The University of Southern Mississippi, Hattiesburg, Mississippi 39406, United States; ‡ Advanced Light Source, 1666 Lawrence Berkeley National Laboratory, Berkeley, California 94720, United States; § Department of Mechanical and Aerospace Engineering, The Hong Kong University of Science and Technology, Clear Water Bay, Kowloon, Hong Kong; ∥ National Synchrotron Light Source II, 8099 Brookhaven National Laboratory, Upton, New York 11973, United States; ⊥ University Grenoble Alpes CEA LETI Grenoble F-38000, France

## Abstract

The backbone conformation of conjugated polymers (CPs) is essential to their performance in electronic applications. Contrast-variation small-angle neutron scattering (CV-SANS) techniques were used to assess the CP’s backbone conformation, which relies on synthesis of deuterated polymers. Such a technique has been proven mature and effective. One drawback is that deuteration labeling might subtly alter polymer’s physical properties due to structural modifications. To address these challenges, we introduce a novel approach utilizing tender X-ray scattering near the sulfur K-edge to distinctly evaluate the backbone versus whole chain conformation for a low-bandgap donor–acceptor CP, poly­[(5,6-difluoro- 2,1,3-benzothiadiazol-4,7-diyl)-*alt*-(3,3‴-dialkyl-2,2′;5′,2″;5″,2‴-quaterthiophen-5,5‴-diyl)] (PffBT4T). For PffBT4T dissolved in trimethylbenzene (TMB), the sulfur K-edge is identified at approximately 2477 eV using near-edge X-ray absorption fine structure spectroscopy (NEXAFS). Tender X-ray scattering conducted at presulfur K-edge and on-sulfur K-edge at elevated temperatures facilitated the distinction between the backbone and whole chain conformations. The results demonstrate that for highly flexible polymer, the backbone’s persistence length could be lower than that of the whole chains, suggesting a more flexible backbone. This rapid, label-free method enhances our ability to characterize CP’s backbone conformation efficiently, offering significant implications for the design and optimization of CPs for advanced electronics.

## Graphical abstract

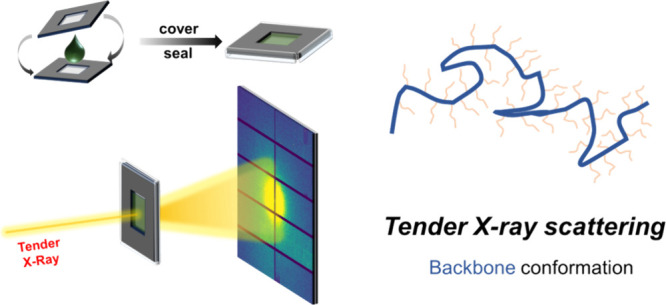

## Introduction

Conjugated polymers (CPs) are crucial for advancing electronic applications, such as thin-film organic field-effect transistors (OFETs), organic solar cells (OSCs), sensors, and light-emitting diodes (LEDs).^1–4^ These polymers typically consist of a conjugated backbone and alkyl side chains, with the backbone primarily responsible for charge transport and thus playing a critical role in device performance.^5–7^ Most of the CP thin films are fabricated using solution-processing methods, such as spin-coating, drop-coating, and blade-coating. Therefore, understanding the conformation of CP’s backbones in solution, especially the rigidity, characterized by persistence length (*L*_p_), is essential for solid-state morphology to optimize device functionality.^8–11^

Solution scattering, particularly small-angle neutron scattering (SANS), is a well-developed method for investigating chain conformation in solution by leveraging scattering contrast between deuterated and nondeuterated components.^12–17^ McCulloch et al. detected the chain shapes of poly­(3-alkylthiophene)­s (P3ATs) in deuterated solvents using SANS and found that the flexibility of the backbone arises from the distribution of *syn* and *anti*-conformations as well as significant backbone torsion in polythiophenes.^12^ Newbloom et al. combined SANS with dielectric spectroscopy and explored the optimum solvent quality for optimizing the conductivity and solubility of various CPs.^15–17^ This technique has also been adapted to study the backbone conformation of CPs by deuteration of the side chains. Cao et al. employed contrast-variation SANS (CV-SANS) experiments on deuterated P3ATs to differentiate backbone and side-chain conformations. By using a mix of protonated and deuterated solvents, they effectively decoupled the scattering signals, revealing that the backbone is more flexible compared to the whole chain.^13^ More recently, they have expanded the study of conformation to a more rigid polymer DPP with deuterated side chains. Combining the CV-SANS, as well as computation modeling, they demonstrated that for semirigid polymer with *L*_p_ ∼ 15 nm, the backbone conformation and whole chain conformation can be different. However, SANS is both time-consuming and costly due to its requirement for deuterated solvents and side chains, extended exposure times, and limited neutron facility availability. Additionally, the deuteration process can potentially alter the properties of the polymers. For example, side-chain deuteration of poly­(3-hexylthiophene) (P3HT) has been reported to significantly reduce the open-circuit voltage due to decreased electronic coupling, the formation of a charge transfer state, and increased electron–phonon coupling.^18,19^ Additionally, backbone deuteration of P3HT has been shown to reduce crystallinity and affect the stability of conjugated polymer crystals.^20^ Furthermore, in a forthcoming study, we found that deuteration also influences the glass transition temperature and melting point. These limitations highlight the need for a more efficient and cost-effective alternative.

Resonant soft X-ray scattering (RSoXS) exploits chemical heterogeneity or interfacial roughness to measure the microstructure or morphology of the material. This method has been extensively used in the field of OSCs to distinguish the contrast between donor and acceptor materials.^21–24^ Various research groups have also applied RSoXS to characterize magnetic and charge heterogeneity,^25,26^ nanoscale structured soft materials,^27,28^ the morphology of organic single or multilayer thin films,^29^ the long-range lateral order in block copolymer films,^30^ and the nanomorphology of conjugated polymers.^31^ The Collins group and the National Institute of Standards and Technology (NIST) group have an extensive review of the RSoXS and reader could refer to that work.^23^ While most resonant scattering studies have been performed on solid thin films, there is significant interest in developing solution-based RSoXS. However, this task is challenging because soft X-rays are easily attenuated by solvents and the cells. To address these challenges, a specialized sample cell adapted from techniques in electron microscopy has been developed to maintain a thin sample thickness and minimize X-ray attenuation. An elegant study by the Collins group has demonstrated that RSoXS can perform label-free contrast control on poly­(ethylene oxide)-*b*-poly­(propylene oxide)-*b*-poly­(ethylene oxide) (PEO-*b*-PPO-*b*-PEO) polymer micelles in water.^24^ The carbon K-edge is located at ∼290 eV. Benefiting from the enhanced contrast to a unique chemical bond at a molecular resonance for RSoXS, PPO shows an extra absorption peak at 287 eV for the methyl group. In this way, PPO contrast with water dominates below the edge, and PEO contrast dominates at the resonance at 289 eV. By performing X-ray scattering at the energy with the greatest contrast, the size of the entire micelle, the core of the micelle, and the aggregation behavior of the micelles can be studied thoroughly.

Recently, a novel approach involving resonant tender X-ray scattering at the sulfur K-edge (∼2477 eV) has been developed, exploiting the unique sulfur atoms at the backbones of CPs.^32–37^ For example, Freychet et al. demonstrated that utilizing polarized tender X-ray scattering at the sulfur K-edge can differentiate different crystalline packing structures which is limited in conventional X-ray diffraction due to the paracrystalline nature of CPs.^32^ Additionally, our group examined the backbone orientation in the crystalline regions of CPs during tensile deformation. It was observed that backbone alignment was limited in glassy CPs but pronounced in viscoelastic CPs.^33,34^ Based on these observations, we hypothesize that utilizing tender X-ray scattering on CP solutions will enable the specific detection and differentiation of backbone conformations.

In this study, we developed a new method to detect the backbone conformation of CPs using tender X-ray scattering near the sulfur K-edge at elevated temperatures. Poly­[(5,6-difluoro- 2,1,3-benzothiadiazol-4,7-diyl)-*alt*-(3,3‴-dialkyl-2,2′;5′,2″;5″,2‴-quaterthiophen-5,5‴-diyl)] (PffBT4T) was selected as the model CP, and trimethylbenzene (TMB) was selected as the solvent. The solution was sealed between silicon nitride (SiNx) windows to perform X-ray scattering. Variable-temperature (VT) small-/wide-angle hard X-ray scattering (hard SAXS/WAXS) was used to determine the optimal temperature (172 °C) for dissolving the CPs. Near-edge X-ray absorption fine structure spectroscopy (NEXAFS) was then conducted to identify the sulfur K-edge. Subsequently, tender X-ray scattering at presulfur K-edge and on-sulfur K-edge energies at 172 °C was performed to differentiate the whole chain and backbone conformations. The 1D scattering profiles were fitted using the flexible cylinder model in the SASview, from which the fitted persistence length (*L*_p_) was obtained for all the energies. Our findings indicate that the *l*_p_ of the backbone is smaller than the one of the whole chains, consistent with SANS measurements reported before.^13^ This new solution-tender X-ray scattering is both rapid (completed within seconds) and label-free, offering an economical and straightforward approach for precise backbone conformation detection.

## Results and Discussion

Resonant tender X-ray scattering at the sulfur K-edge is used to investigate the structure of sulfur-containing CPs. Leveraging the properties of sulfur atoms, this technique enhances contrast between the sulfur components and their nonsulfur counterparts. In this study, we selected PffBT4T as a model CP because its sulfur atoms are exclusively located within the backbone (Figure 1). This unique feature allows for a targeted examination of the backbone conformation when PffBT4T is in solution, which is essential for understanding its solid-state morphology and optimizing device performance. Previous studies have shown that PffBT4T can be fully dissolved into single chains at elevated temperatures, enabling the investigation of single-chain backbone conformation.^14^ Trimethylbenzene (TMB) and SiNx were selected as the solvent and sample holder, respectively, due to the absence of sulfur atoms and low absorption. The solution was encapsulated between two SiNx windows. The reliability of this sample preparation method has been confirmed by the hard SAXS of a polystyrene solution. Details can be found in the Supporting Information (Figures S1–S3).

**Figure 1:**
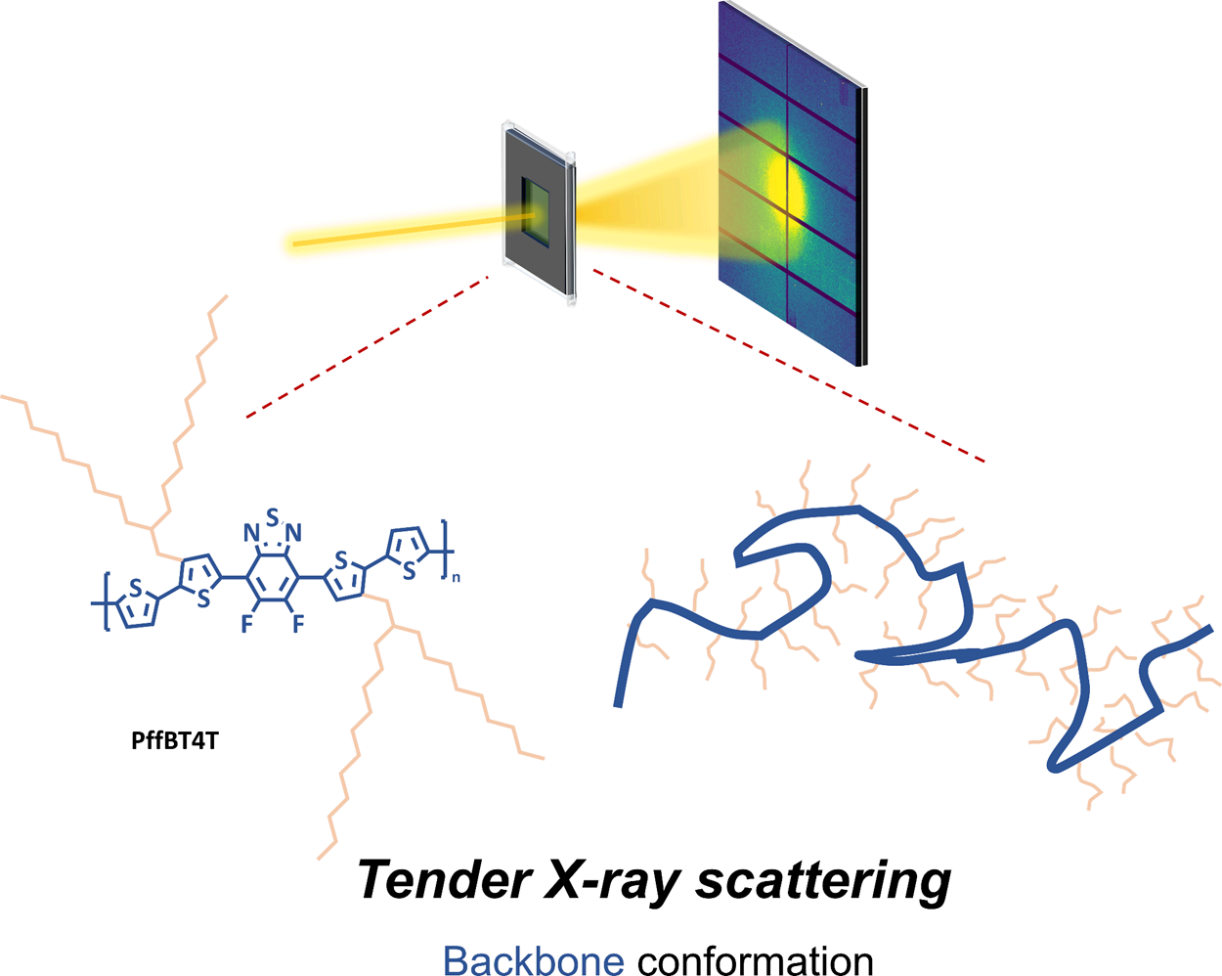
Schematic illustration of the backbone conformation study of CPs using resonant tender X-ray scattering. The figure includes the chemical structure of model polymer PffBT4T, highlighting that sulfur atoms are exclusively located in the backbone.

The chain conformation and aggregation behavior of PffBT4T are sensitive to temperature.^14^ At room temperature, strong interchain interactions promote aggregation in solution. As the temperature increases, these aggregates dissolve, allowing single polymer chains to form. *In situ* VT-hard SAXS/WAXS measurements on PffBT4T solution were performed from 25 to 200 °C, confirming that 172 °C is the optimum temperature (Figures S4–S6). This optimal temperature is slightly higher than previously reported, likely due to heat insulation between the SiNx window and the heating stage.^14^ Similar experiments using tender X-ray scattering produced results nearly identical to those from hard X-ray measurements (Figure S7).

NEXAFS spectroscopy was conducted across the sulfur K-edge to identify energies with the maximum scattering contrast. The contrast in resonant tender X-ray scattering arises from the difference in refractive index between the sulfur-containing backbone and the nonsulfur components (solvent and side chains). It is described by its real and imaginary components, *n*(*E*) = 1 – δ­(*E*) + *i*β­(*E*). The β­(*E*) of PffBT4T was measured using X-ray fluorescence data collected during the resonant scattering experiments, and δ­(*E*) was calculated from β­(*E*) using the Kramers–Kronig transformation (Figure 2a).^38^ The β­(*E*) and δ­(*E*) of the solvent TMB were calculated based on the Henke X-ray Database. The contrast between PffBT4T and TMB peaks at 2477 eV enhanced the contrast from the backbone (Figure 2b). Additionally, the contrast between various polymer chain orientations vs the beam polarization was measured to understand the influence of local polymer chain anisotropy on the scattering contrast. It was found the overall contrast, even averaged across all orientations, is strongly dominated by the contrast between the backbone aligned parallel to the beam polarization (Figure S8). The prominent feature in the *f*_*z*_″ spectrum can be associated with 1s (C–C) σ* transitions, consistent with previous findings by McNeil et al.^32^ Therefore, the scattering at the sulfur K-edge is suitable to investigate the PffBT4T backbone conformation. Conversely, for off-resonant X-ray scattering, the scattering contrast arises from electron density differences between the molecules and the solvent, similar to the case for conventional hard X-ray scattering. Based on the calculation, the contrast between both backbone and side chain showed significant difference at off-edge, indicating the efficiency of whole chain study (Supporting Information).

**Figure 2:**
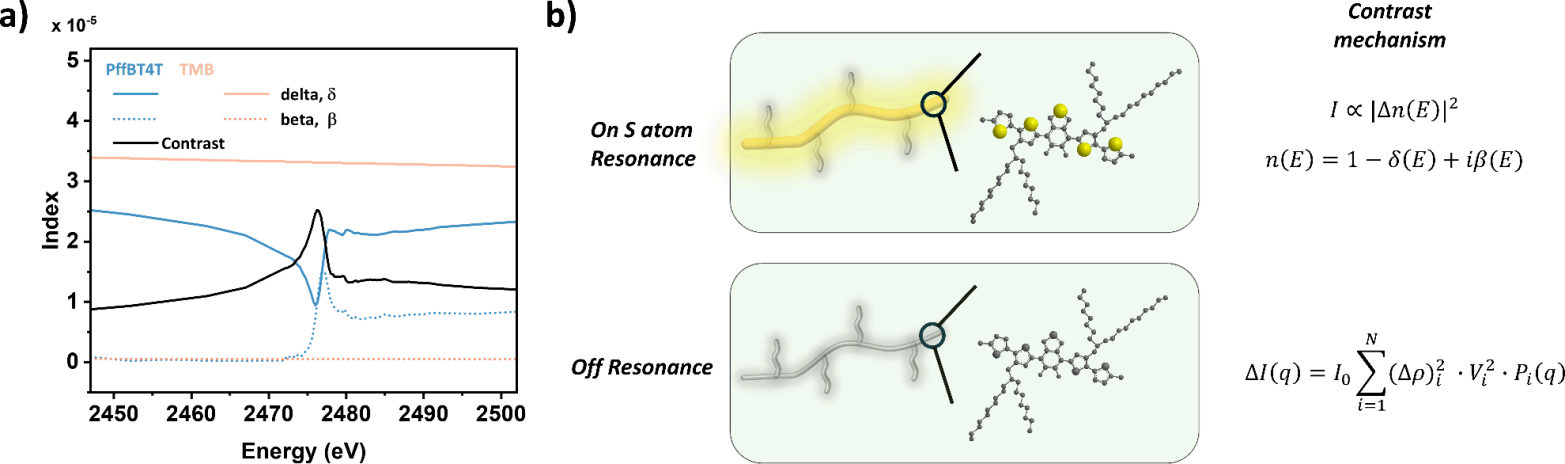
(a) Optical constants, delta (δ), and beta (β) as a function of energy for PffBT4T solutions (blue) and solvent TMB (pink). Contrast between PffBT4T solution and TMB (black). (b) Chemical structure of CP, PffBT4T with the sulfur atoms highlighted by Tender X-ray. Contrast mechanism for X-ray scattering at pre- and on-sulfur K-edge.

Tender X-ray scattering measurements at energies around the sulfur K-edge (2470–2480 eV) at 172 °C were conducted to investigate the backbone conformation. 2D scattering patterns and 1D profile of PffBT4T solutions are shown in Figures 3a and S9. Due to weak signals and detector noise, we applied careful masking and flat-field corrections in the 2D scattering pattern before reducing to the 1D profile. To isolate the scattering signal from the PffBT4T polymer chains, the 1D profile was then subtracted from the solvent TMB (Figures S10 and S11). Since the PffBT4T solution and the TMB solvent were contained in different SiNx windows, resulting in variations in scattering volume, a subtraction factor was applied during data processing. Various subtraction factors have been tried, and the results are displayed in Figures S12–S19. When a higher subtraction factor is used, the high-*q* region is over subtracted and smaller than 0. To prevent oversubtraction in the high-q region, a consistent factor of 1.7 was applied across all energies. While this factor may not provide perfect accuracy, it allows for a reasonable data comparison. The further development of flow cells for tender X-ray scattering could help in future studies. Detailed data processing can be found in Supporting Information.

**Figure 3:**
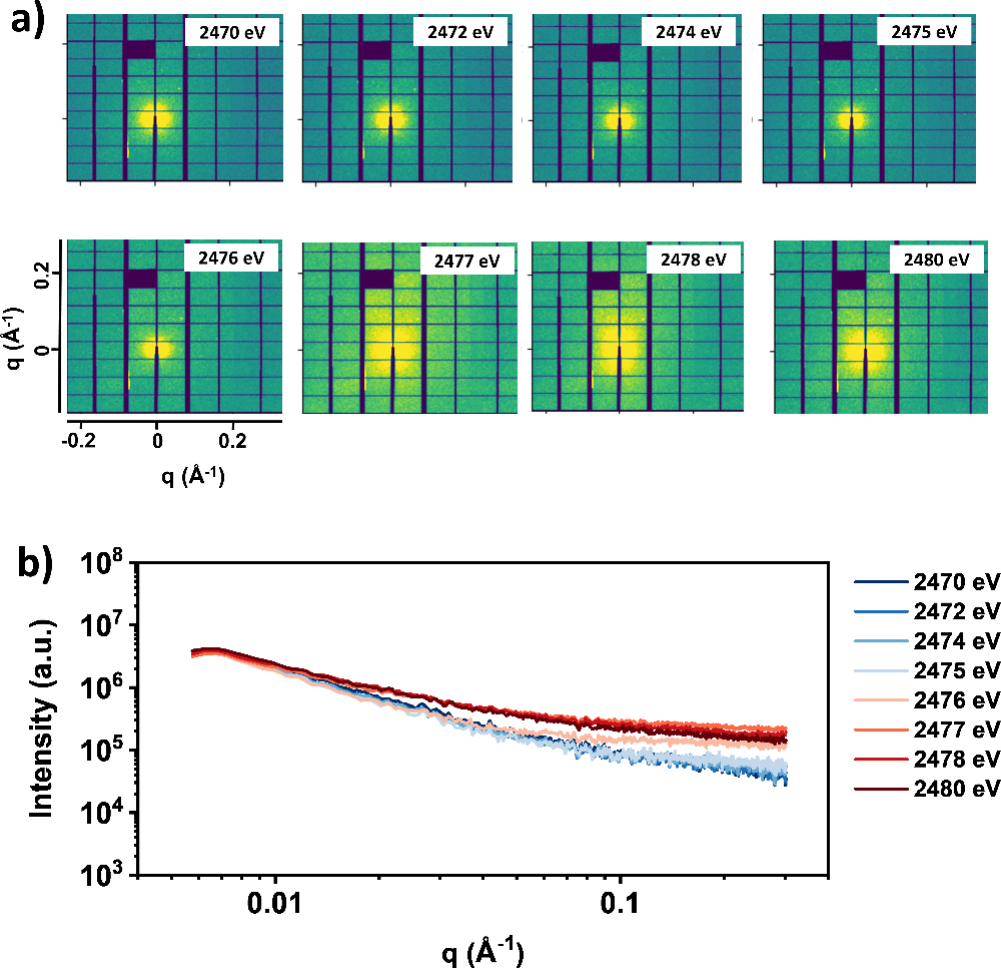
(a) 2D scattering patterns and (b) 1D tender X-ray scattering profile of PffBT4T at different energies (2470–2480 eV) near the sulfur K-edge at 172 °C after background subtraction from the solvent, TMB, without fluorescence correction.

The subtracted 1D profiles for PffBT4T are summarized in Figure 3b. From 2476 to 2477 eV, an increase in intensity was observed in both the 2D patterns and 1D profiles of PffBT4T. This increase is attributed to X-ray fluorescence from sulfur atoms. Integration of the subtracted 1D data over the entire *q*-range showed good correlation with results of NEXAFS, which further confirmed the contrast was originated from the sulfur atom in PffBT4T (Figure S20). Conversely, the intensity of TMB remained constant across energies due to the lack of sulfur atoms.

The subtracted 1D scattering profiles at all energies were then fitted using the Flexible Cylinder Model (FCM), commonly employed for semiflexible CP chains, to determine the persistence lengths *(L*_p_) for both the backbone and the entire chain. The *L*_p_ quantifies the polymer chain’s rigidity, which is crucial for designing CPs with enhanced electrical properties.^39^ It should be noted that ambiguity may arise in the contrast during FCM fitting as the effective scattering length density (SLD) or optical properties of the cylinders may not be uniform in the experiment, even when using circularly polarized light. The contour length (*L*_c_) was fixed at 200 nm, calculated from the molecular weight of PffBT4T, and the cylinder radius was set at 0.94 nm, based on previous SANS measurements.^14^ The original, fitted, and residual curves at all energies are provided in Figures S21–S28. To confirm that fixing the contour length does not influence the *L*_p_ fitting results, we used SASView to simulate scattering curves with varying contour lengths (200, 250, and 300 nm), while keeping the *L*_p_(3 nm) and cylindar radius (0.94 nm) constant (Figure S29). The simulations show that changes in *L*_c_ affect only the low-*q* regime of the scattering curve and do not impact the mid-*q* region used for fitting the *L*_p_. This demonstrates that the assumed *L*_c_ (or equivalently, molecular weight) does not significantly influence the persistence length fitting results.

At off-edge energies (2470–2476 eV), the *L*_p_ was approximately 3.0 nm, representing the rigidity of the entire polymer chain (Figure 4, Table 1). At on-edge energies (2477–2480 eV), *L*_p_ decreased to about 2.0 nm, indicating that the backbone alone is more flexible than the whole chain. These results are consistent with our previous findings on flexible P3AT polymer, where the backbone demonstrated greater flexibility relative to the entire polymer chain.^13,40^ This behavior was supported by coarse-grained molecular dynamics (CG-MD) simulations. Based on our previous result from all-atomistic molecular dynamics (AA-MD) simulation, the flexibility of the backbone of PffBT4T was supposed from the C–C bond.^41^

**Figure 4:**
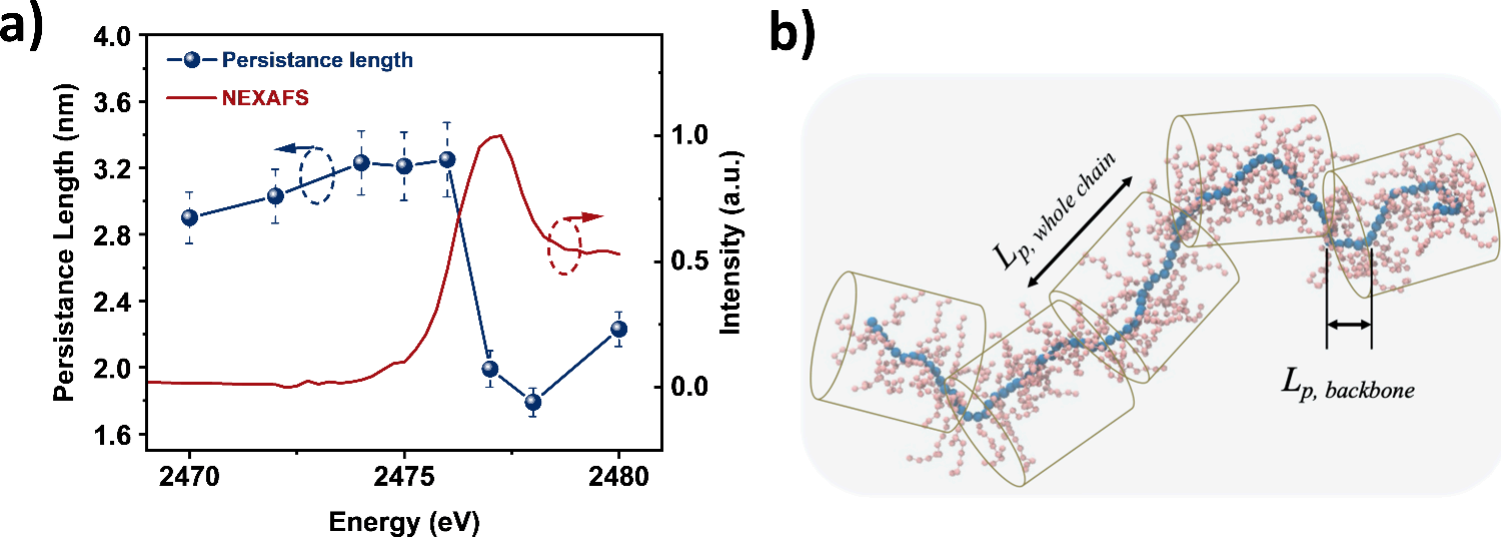
(a) Summary of the fitted persistence length for PffBT4T across different energies around the sulfur K-edge and NEXAFS spectrum. (b) Schematic of the PffBT4T polymer chain with whole chain persistence length longer than backbone.

**Table 1:** Parameters Obtained from Fitting the Tender Wide-Angle X-ray Scattering (WAXS) Data Using the Flexible Cylinder Model

energy (eV)	*L*_c_ (nm)	*L*_p_ (nm)	fitting error (nm)	radius (nm)
2470	200	2.90	0.15	0.94
2472	200	3.03	0.16	0.94
2474	200	3.23	0.19	0.94
2475	200	3.21	0.21	0.94
2476	200	3.25	0.22	0.94
2477	200	1.99	0.11	0.94
2478	200	1.79	0.09	0.94
2480	200	2.23	0.10	0.94

The Flexible Cylinder Model is *P*(*q*) = scale⟨*F* ^2^⟩/*V* + background. During the fitting process, the fluorescence signal from sulfur was treated as an *q*-independent constant background, which does not affect the fitting result. To verify that fluorescence did not significantly affect the results, we fitted the 1D scattering profile again after subtracting the fluorescence contribution. We estimated the fluorescence signal by the intensity difference between energies E = 2470 eV and *E* = 2477 eV in the high-*q* region (*q* ∼ 0.3 Å^–1^), where the contribution from scattering is minimal (as the scattering intends to decay with increasing *Q* and will fall below background in high *q*) and the signal is dominated by fluorescence. The 1D scattering profile of PffBT4T at 2477 eV after fluorescence subtraction is shown in Figure S30. The fitted persistence length *L*_p_ of 1.99 nm closely matches the value obtained before fluorescence correction, confirming that fluorescence does not significantly affect the fitting results. The fitted constant background at different energies, as shown in Figure S31, aligns well with the NEXAFS data. This further confirms that the fluorescence signal was appropriately considered during the fitting.

## Experimental Section

### Materials

Poly­[(5,6-difluoro-2,1,3-benzothiadiazol-4,7-diyl)-*alt*-(3,3‴-di­(2-nonyltridecyl)-2,2′;5′,2″;5″,2‴-quaterthiophen-5,5‴-diyl)] (PffBT4T-C9C13) was purchased from Ossila. Anhydrous trimethylbenzene (TMB) was ordered from Sigma-Aldrich. SiNx window (thickness: 1000 nm) was obtained from Norcada. Solvent-resistant epoxy was purchased from Gorilla. All chemicals were used as received without further purification.

### Sample Preparation for Tender X-ray Scattering

PffBT4T was dissolved in TMB at a concentration of 10 mg/mL and heated at 80 °C overnight to prepare the PffBT4T solution. SiNx was employed as the sample holder.

Proper sample preparation is required to ensure compatibility with the vacuum experimental conditions and to achieve optimal X-ray scattering signals. For sample assembly, we deposited 1.70 μL of the hot PffBT4T solution onto a SiNx window and promptly placed another SiNx window on top to prevent the solution from drying. The edges were sealed with epoxy to prevent leakage. This sealing method effectively prevented material loss in the high-vacuum chamber (between 10^–3^ and 10^–6^ Torr) at temperatures ranging from 25 to 200 °C during extended measurements. The approximate 200 μm gap between the two SiNx windows allows tender X-rays to pass through the solution and both windows without excessive attenuation. Detailed sample preparation method and reliability study can be found in the Supporting Information.

### Hard/Tender X-ray Scattering

X-ray scattering measurements were performed on a PffBT4T polymer solution in TMB (10 mg/mL). Hard/tender X-ray scattering was performed at the Soft Matter Interfaces (SMI) beamline (Beamline 12-ID) at the National Synchrotron Light Source II in vacuum using a transmission geometry. Hard X-ray scattering patterns were measured at an energy of 16.1 keV, recorded on a Pilatus 1 M detector. VT-hard X-ray scattering was performed from 25 to 200 °C. Tender X-ray scattering patterns were measured as a function of energy by varying the photon energy between 2445 and 2500 eV, recorded on a Pilatus 300 KW detector, consisting of 0.172 mm square pixels in a 1475 × 195 array, mounted at a fixed distance of 0.28 m from the sample position. The sample was shifted laterally for each measurement to avoid beam damage. The spot size at the sample was 20 μm by 200 μm. Data processing includes (1) data reduction from 2D pattern to 1D profiles, (2) removing scattering from solvent, and (3) fitting the data in flexible cylinder model in SAS view, with consideration of fluorescence as a constant *q*-independent signal. Detailed data reduction and analysis can be found in Supporting Information.

### Safety Statement

No unexpected or unusually high safety hazards were encountered.

## Conclusions

In summary, we have developed a novel method using tender X-ray scattering near the sulfur K-edge to detect and differentiate the backbone conformation from the whole chain conformation of conjugated polymers. A solution of PffBT4T in TMB was selected as the model CP and sealed between the SiNx windows. NEXAFS was conducted to identify the sulfur K-edge. Subsequent tender X-ray scattering at presulfur-edge and on-sulfur-edge energies at elevated temperatures allowed for the differentiation of whole chain and backbone conformations. The 1D scattering profiles were analyzed by using the flexible cylinder model. The backbone’s persistence length was smaller than that of the whole chains, indicating a more flexible backbone. Therefore, by combining on-edge and off-edge scattering data, we effectively distinguished between the backbone and whole-chain conformations of PffBT4T, demonstrating the utility of resonant tender X-ray scattering for such analyses. This new method provides a rapid (completed within seconds), label-free approach for detecting backbone conformation, which is crucial for guiding the future design of CPs.

## Supplementary Material



## Data Availability

All code used in this project are available at the public GitHub repository https://github.com/feibywang/Resonant-Tender-X-Ray-Scattering-for-Disclosing-the-Backbone-Conformation-of-Conjugated-Polymers.

## Notes

Sample preparation and reliability study; VT-small/wide angle hard X-ray scattering (SAXS/WAXS) of PffBT4T solution; contrast calculation at off-edge; detailed tender X-ray scattering data analysis; and supporting figures (Figures S1–S29) (PDF)
