# Supplementary material for: Resonant Tender X‑ray Scattering for Disclosing the Backbone Conformation of Conjugated Polymers
Source: Macromolecules. 2025 Jun 26;58(13):6865–71. doi: 10.1021/acs.macromol.4c03107 (PMC12257596; doi:10.1021/acs.macromol.4c03107)
Supplement: Supplementary file 1 [file ma4c03107_si_001.pdf]

Supporting Information

Resonant Tender X-ray Scattering for Disclosing the backbone conformation of conjugated Polymers

Yunfei Wang,[1,2,‡] Ka Hung Chan,[2,3,‡] Guillaume Freychet,[4,5,‡] Patryk Wąsik,[4] Song Zhang,[1] Zhiqiang Cao,[1] Xiaodan Gu[1,*]

[1] School of Polymer Science and Engineering, Center for Optoelectronic Materials and Devices, the University of Southern Mississippi, Hattiesburg, MS 39406, USA

[2] Advanced Light Source, Lawrence Berkeley National Laboratory, Berkeley, California, USA

[3] Department of Mechanical and Aerospace Engineering, The Hong Kong University of Science and Technology, Clear Water Bay, Kowloon, Hong Kong

[4] National Synchrotron Light Source II, Brookhaven National Laboratory, Upton, NY 11973, USA

[5] University Grenoble Alpes CEA LETI Grenoble F-38000, France

‡ These authors contribute equally to this work.

Sample preparation and reliability study

PffBT4T was dissolved in TMB at a concentration of 10 mg/mL and heated at 80 °C overnight to prepare PffBT4T solution.

Trimethylbenzene (TMB) was selected as the solvent due to the absence of sulfur atoms, high boiling point (169 °C), excellent solubility for CPs, and low absorption in the tender X-ray energy range compared to chlorine-containing solvents like chlorobenzene. The absence of sulfur atoms in TMB enables enhances contrast between the PffBT4T backbone and the solvent, facilitating detailed studies of backbone conformation (**Figure S1a**). Its relatively high boiling point of 169°C allows for experiments at elevated temperatures, while its excellent solubility for CPs ensures thorough dissolution into single chains. Moreover, TMB exhibits lower absorption in the tender X-ray energy range compared to commonly used chlorine-containing solvents for CPs such as chlorobenzene making it suitable for tender X-ray scattering (**Figure S1a and S1b)**. Silicon nitride (SiNx) window was employed as the sample holder due to its low absorption in the tender X-ray scattering range, further optimizing experimental conditions (**Figure S1c**)

Proper sample preparation is required to ensure compatibility with vacuum experimental conditions and to achieve optimal X-ray scattering signals. For sample assembly, we deposited 1.70 μL of the hot PffBT4T solution onto a SiNx window and promptly placed another SiNx window on top to prevent the solution from drying, forming a "sandwich" structure. The edges were sealed with epoxy to prevent leakage (**Figure S2**). This sealing method effectively prevented material loss in the high-vacuum chamber (between 10^{-3} and 10^{-6} Torr) at temperatures ranging from 25 to 200 °C during extended measurements. The approximate 200 μm gap between the two SiNx windows allows tender X-rays to pass through the solution and both windows without excessive attenuation**.**

To verify the reliability of the sample preparation method, we initially prepared a solution of well-studied polymer, polystyrene (molecular weight ~ 173 kDa, PS173k) in TMB, for small-angle hard X-ray scattering (hard SAXS). The SAXS profile (scattering intensity vs. scattering vector plot, **Figure S3**) of the PS solution was subsequently fitted using the Debye Gaussian coil model, which is suitable for monodisperse polymer coils. The fitted radius of gyration (R_g) was 82.77±7.25 Å. For a PS of 173 kDa, the theoretical R_g is 111 Å in a good solvent and 36 Å in a theta solvent. Consequently, TMB acts as an intermediate solvent, leaning more towards the characteristics of a good solvent. This finding substantiates the success of our sample preparation method.

VT-small/wide angle hard X-ray scattering (SAXS/WAXS) of PffBT4T solution

To determine the optimal temperature for studying single-chain conformations, we conducted *in situ* variable-temperature SAXS and wide-angle X-ray scattering (WAXS) experiments from 25 °C to 200 °C.

The exact same samples were used for variable-temperature (VT) hard/tender X-ray scattering to ensure consistency between all the scattering experiments. The experimental setup is shown in **Figure S4**. Samples between the SiN window with high temperature epoxy glue were mounted on a sample bar and placed on a heating stage controlled by temperature controller. At each set temperature, the system was allowed to equilibrate for 10 minutes before data collection.

The WAXS data showed a (100) lamellar peak at q ~ 0.22 $Å^{-1}$ at lower temperatures (25, 55, and 109 °C), indicating significant crystallite aggregation **(Figures S4)**. This peak diminished substantially at 158 °C, suggesting that the aggregates were dissolving. Similarly, the SAXS intensity decreased significantly at higher temperatures due to the reduction of the structure factor associated with aggregates **(Figures S5)**. At 172 °C and above, the intensity stabilized, indicating complete dissolution into single chains. Therefore, 172 °C was selected as the optimize temperature for single chain study.

Contrast calculation at off-edge

In small-angle X-ray scattering (SAXS), the total scattered intensity can be decomposed into coherent part and the incoherent part. The incoherent light scattering primarily includes background noise, such as fluorescence, which is independent of the scattering vector q.

$$I = I_{coh} + I_{incoh}$$

The coherent scattering provides information about the structural properties of the molecules in the solution. For a molecule dispersed in a solvent, the coherent scattering intensity can be expressed as:

$$I_{coh}(q) = |\rho_m - \rho|^2 V_m^2 \langle F_m|q|^2 \rangle$$

Where the ρ_m is the scattering length density (SLD) of the molecule and

ρ is the SLD of the solvent,

V_m is the volume of the molecule,

$F|q|$ is the form factor of the molecule.

The scattering length density ρ is a complex quantity comprising both real and imaginary components, which contribute to the overall contrast in SAXS measurements:

$$Re[\rho] = \delta$$

$$Im[\rho] = \beta$$

When considering a solution containing two distinct molecular components, such as a backbone (B) and a side chain (S), the coherent scattering intensity becomes:

$$I_{coh}(q) = |\rho_B - \rho|^2 V_B^2 \langle F_B|q|^2 \rangle + |\rho_S - \rho|^2 V_S^2 \langle F|q|^2 \rangle + 2|\rho_B - \rho||\rho_S - \rho| \langle F_{BS}|q| \rangle$$

The scattering length density of the solvent can be estimated using its molecular weight, and density. For TMB (1,3,5-Trimethylbenzene), the calculated values are:

$$\delta = 3.331 \times 10^{-5}$$

$$\beta = 5.098 \times 10^{-7}$$

Assuming a bulk density of 1 g/cm^3 for the side chain, the SLD of the side chain is determined to be:

$$\delta_S = 3.952 \times 10^{-5}$$

$$\beta_S = 5.524 \times 10^{-7}$$

Assuming a bulk density of 1 g/cm^3 for the backbone, the SLD of the side chain is determined to be:

$$\delta_S = 2.746 \times 10^{-5}$$

$$\beta_S = 8.309 \times 10^{-7}$$

This indicates both the backbone and side chain have a significant contrast in TMB.

Detailed Tender X-ray Scattering Data Analysis

The data processing follows: 1) data reduction from 2D to 1D, 2) then remove scattering from solvent, 3) follow by fitting the data in flexible cylinder model in SASview, with consideration of fluorescence as a constant q-independent signal.

Step 1: Reducing 2D scattering from the detector image into 1D scattering I vs Q curve

Due to weak signals and detector noise, we applied careful masking and flat-field corrections during the 1D profile reduction. The scattering profile is reduced from 2D pattern using the python package smi-analysis. (https://github.com/NSLS-II-SMI/smi-analysis). The masking is done in an image by image case. 2D patterns and 1D scattering profile of PffBT4T solutions were shown in **Figure 3a and Figure S7**, using approached described above.

Step 2: Removing solvent scattering from the sample scattering

To isolate the scattering signal from the PffBT4T polymer chains, the 1D profile of PffBT4T in TMB was subtracted from the scattering signal solvent TMB.Additional data for solvent scattering removal at other X-ray energies is provided in the supporting information (**Figures S7–S17**) for those interested in further examination. Since the PffBT4T solution and the TMB

solvent were placed in different custom-made sample cells, constructed with individual SiNx windows glued with homemade epoxy, variations in scattering volume occurred. To account for these differences, a scaling factor was applied during data processing. Various scaling factors, ranging from $f = 1.1$ to 2.9, were tested at all X-ray energies using a custom Python script (code available at https://github.com/feibywang/Resonant-Tender-X-Ray-Scattering-for-Disclosing-the-Backbone-Conformation-of-Conjugated-Polymers). The results of this analysis are presented in Figure R2. To prevent over-subtraction in the high- q region, a consistent scaling factor of $f = 1.7$ was applied across all energies. While this factor may not provide perfect accuracy, it ensures reasonable data comparison. Future studies could benefit from the development of a flow cell specifically designed for tender X-ray scattering, enabling more precise solvent signal subtraction and reducing variability, similar to the flow cell that has been developed for RSOXS beamline in ALS.

Step 3: Fitting the 1D scattering data in the SASview

The subtracted 1D scattering profiles at all energies were fitted using the Flexible Cylinder Model, a standard approach for analyzing semi-flexible conjugated polymer (CP) chains, to estimate the persistence lengths (L_p) of both the backbone and the entire chain. The contour length was fixed at 200 nm, calculated based on the molecular weight of PffBT4T, while the cylinder radius was set at 0.94 nm, as determined from previous small-angle neutron scattering (SANS) measurements. The scattering data fitting was performed using SASview software.

The original, fitted, and residual curves for all energies are provided in **Figures S19–S26**, with an example shown in Figure R3 for E = 2477 eV. As requested by the reviewer, the residuals of the fittings have also been included for clarity.

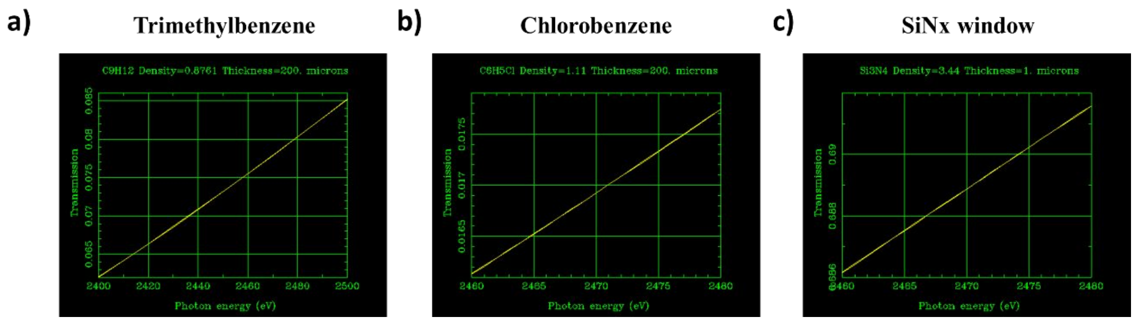

Figure S1. Transmission of (a) trimethylbenzene (TMB, 200 um thickness, the thickness of SiNx window frame) (b) chlorobenzene (common solvent for conjugated polymers, 200 um thickness, the thickness of SiNx window frame) and (c) SiNx window (1000 nm) near the sulfur K-edge.

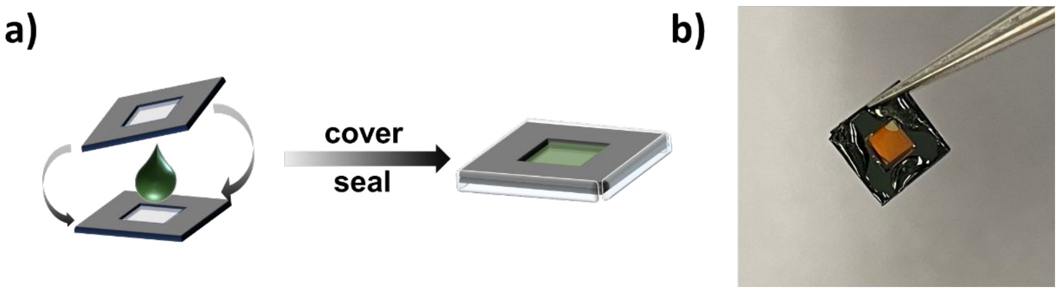

Figure S2. a) Schematic of solution sample preparation for tender X-ray scattering. b) Representative optical images of solution samples between sealed SiNx windows.

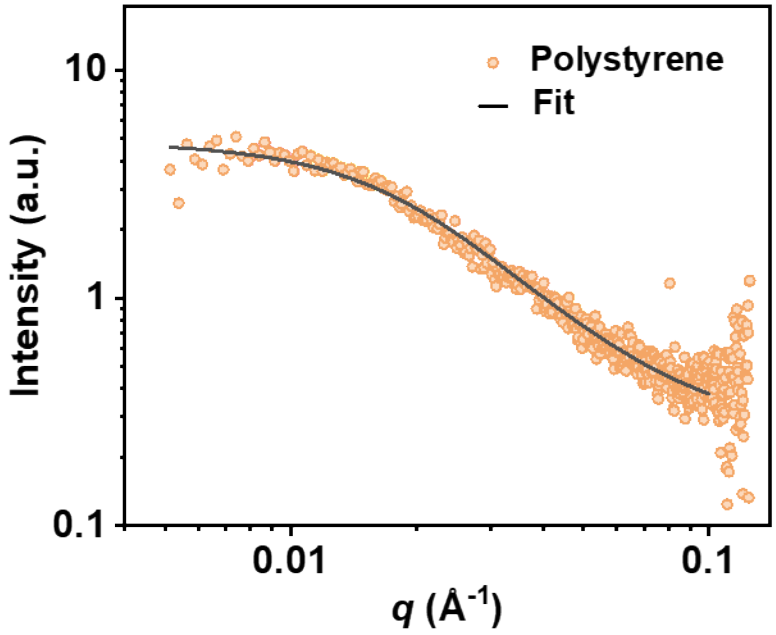

Figure S3. Scattering profiles for polystyrene solution in toluene. Solid lines correspond to the best fit to the Debye Gaussian coil model.

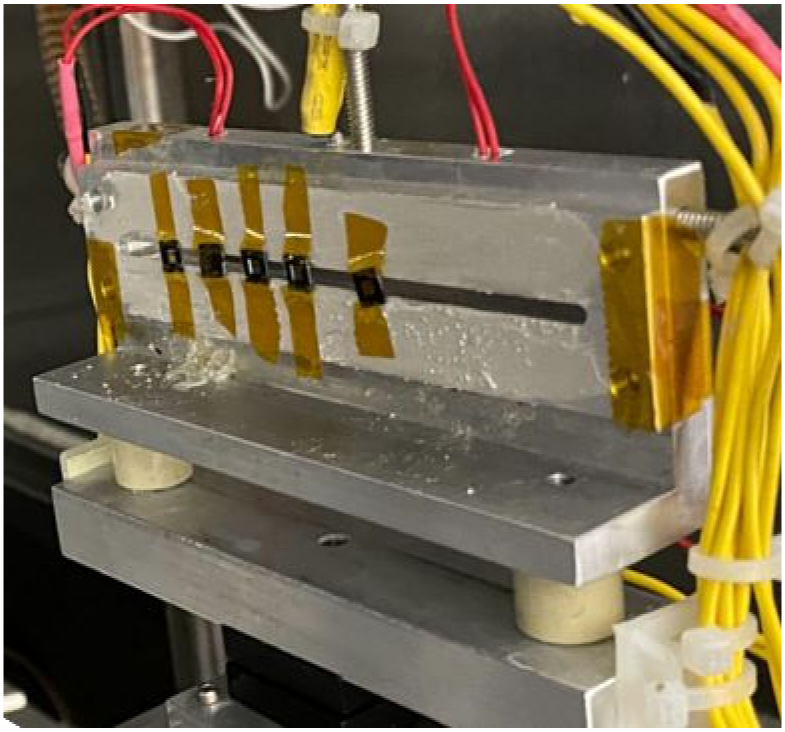

Figure S4. VT X-ray scattering experiment setup.

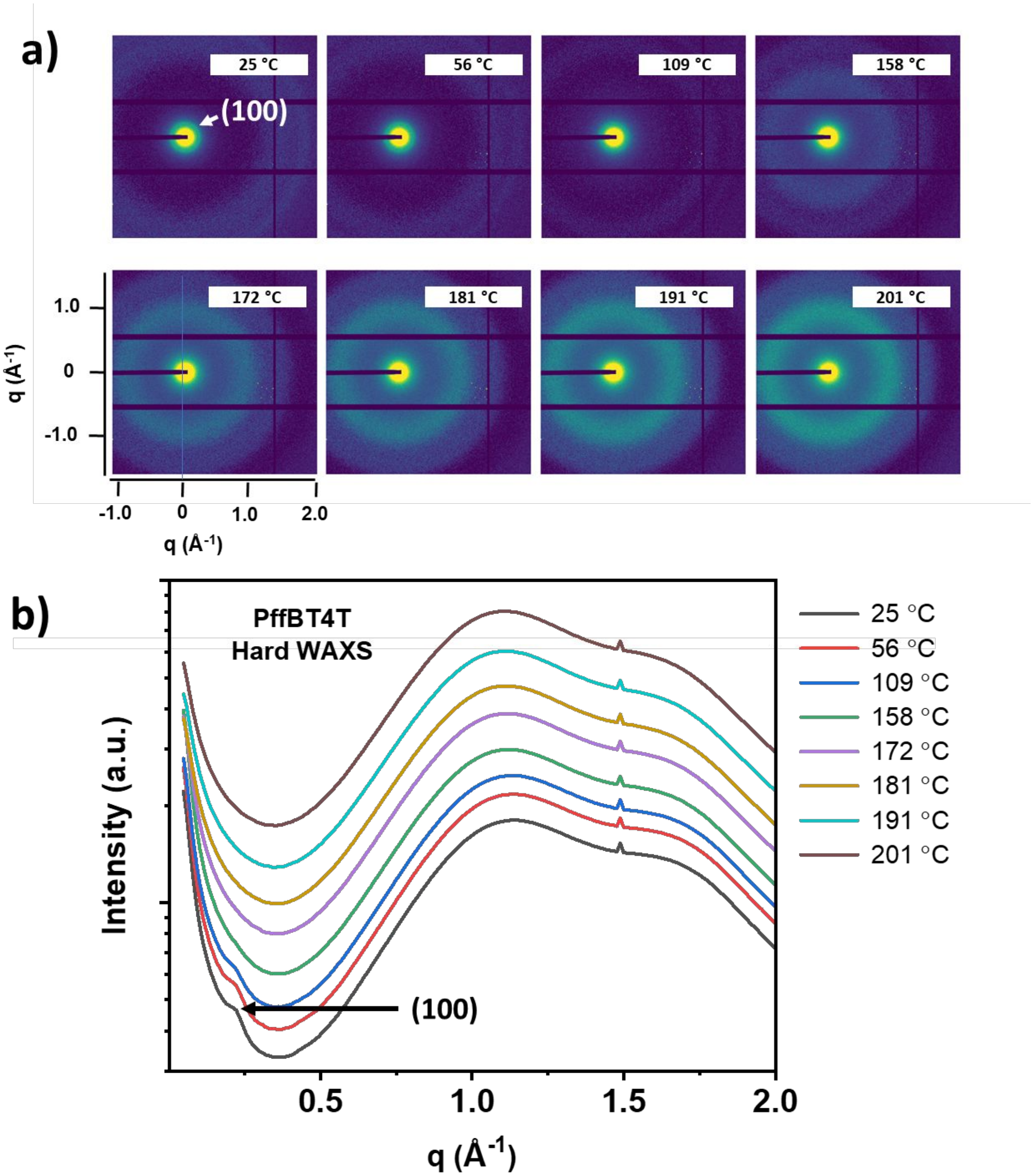

Figure S5. (a) 2D patterns and (b) 1D line-cut of PffBT4T solutions at different temperatures from wide-angle hard X-ray scattering.

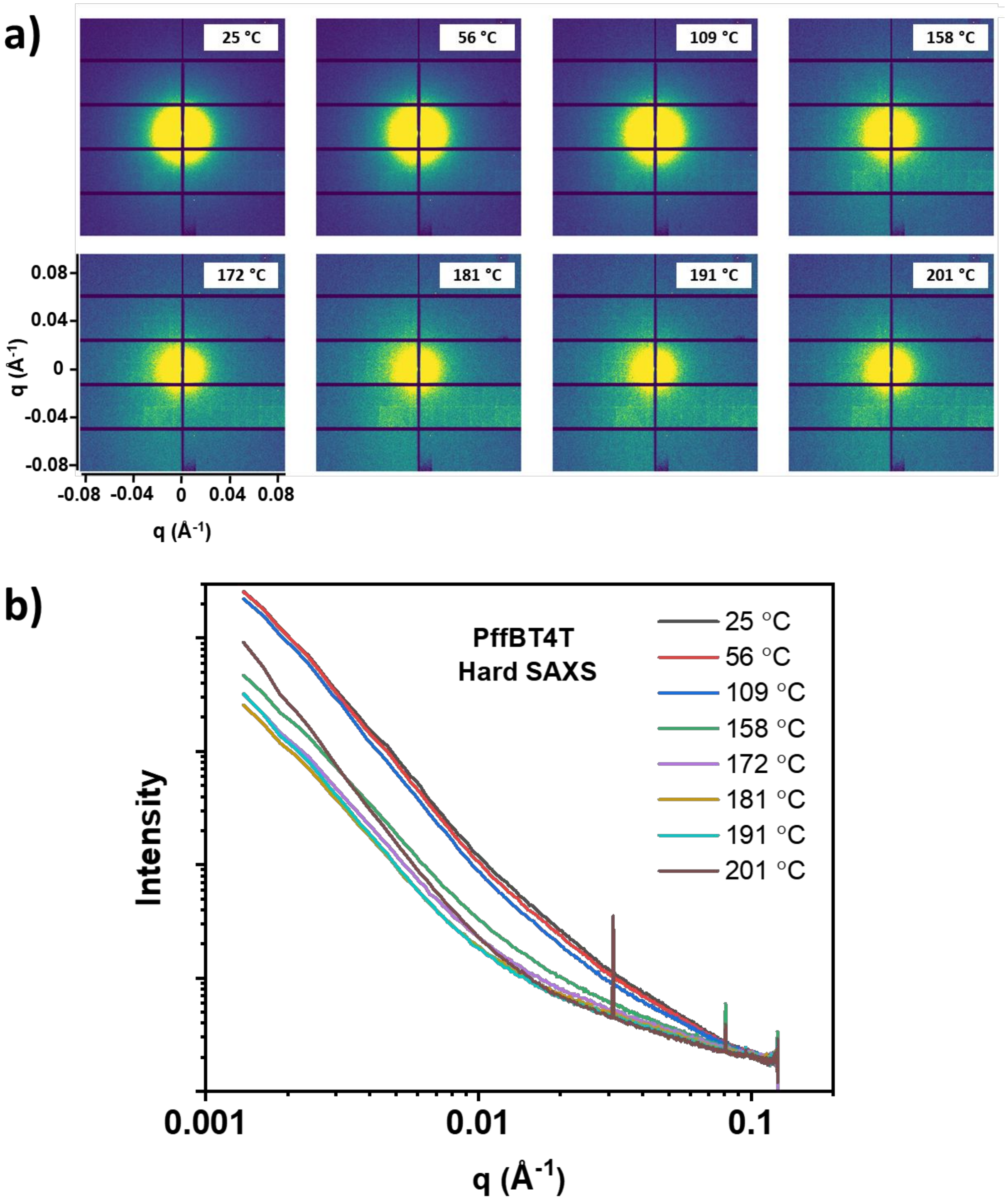

Figure S6. (a) 2D patterns and (b) 1D line-cut of PffBT4T solutions at different temperatures from small-angle hard X-ray scattering.

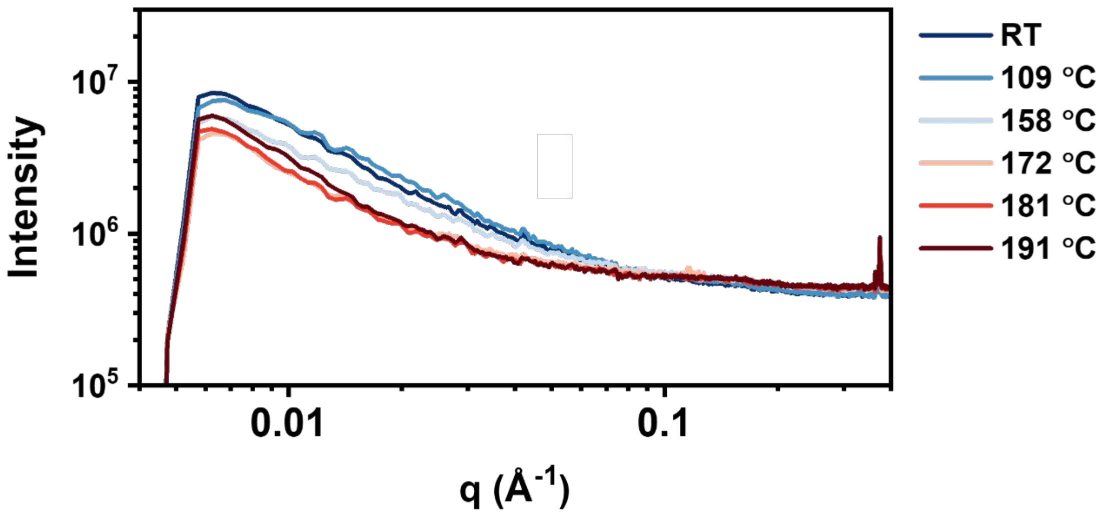

Figure S7. Tender X-ray scattering profile of PffBT4T solutions at different temperatures and energy of 2477 eV. Strong scattering intensity at low scattering vectors range (q ~ 0.006-0.05 Å^{-1}) indicated aggregations at room temperature (RT) and 109°C. The intensity began to decrease at 158°C due to a reduction in the structure factor, signaling the dissolution of aggregates. The intensity further decreased at 172°C and stabilized at higher temperatures, indicating a complete dissolution to single chain at 172°C. Consequently, 172°C was selected as the temperature for further single chain conformation studies.

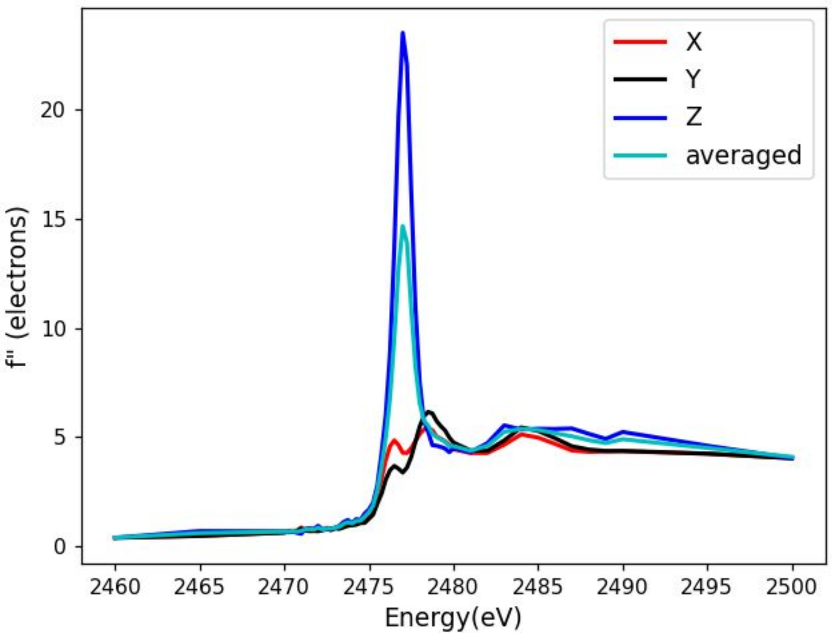

Figure S8. Imaginary scattering factor spectrum *f''* versus energy around sulfur K-edge. With *fx''* being applicable when the X-ray polarization is parallel to the lamellar stacking direction, *fy''* being applicable when the X-ray polarization is parallel to the π-π stacking direction, and *fz''* being applicable when the X-ray polarization is parallel to the backbone stacking direction.

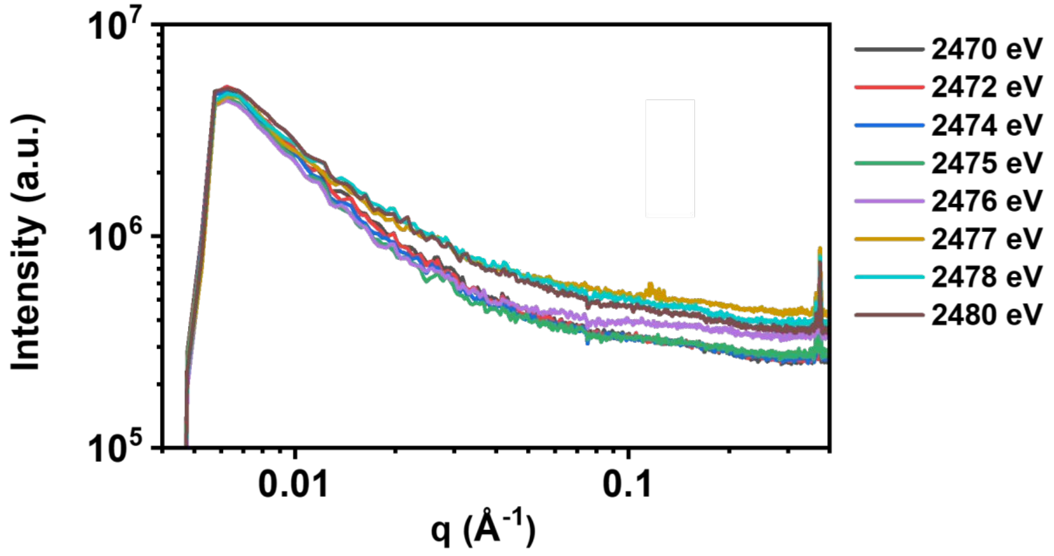

Figure S9. Original tender X-ray scattering profile of PffBT4T solutions at different energies (2470-2480 eV) near sulfur K-edge from at 172 °C without background subtraction from the solvent, TMB.

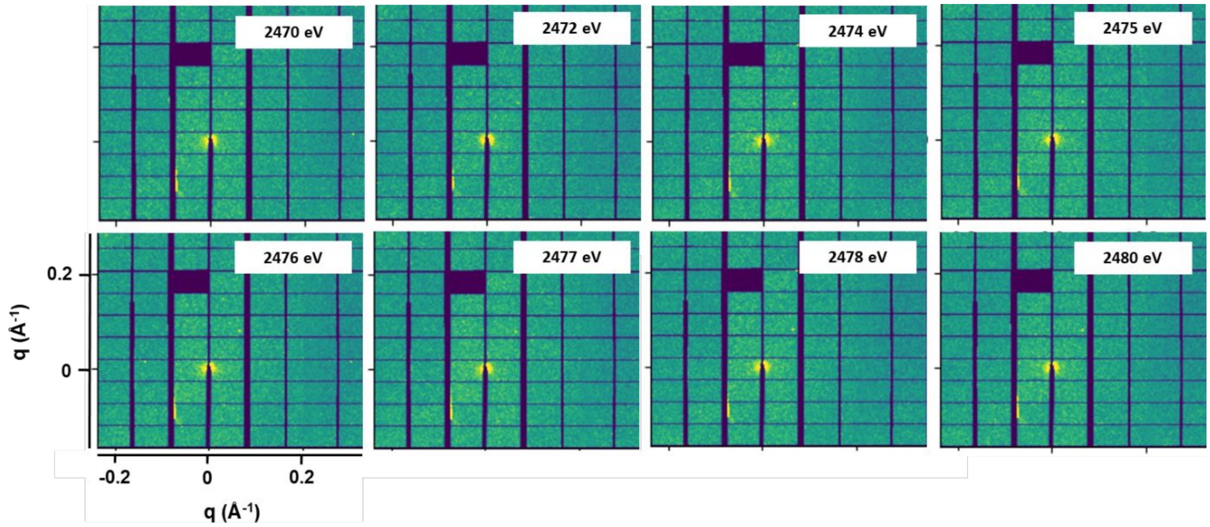

Figure S10. 2D patterns of solvent, TMB, at different energies (2470-2480 eV) near sulfur K-edge at 172 °C.

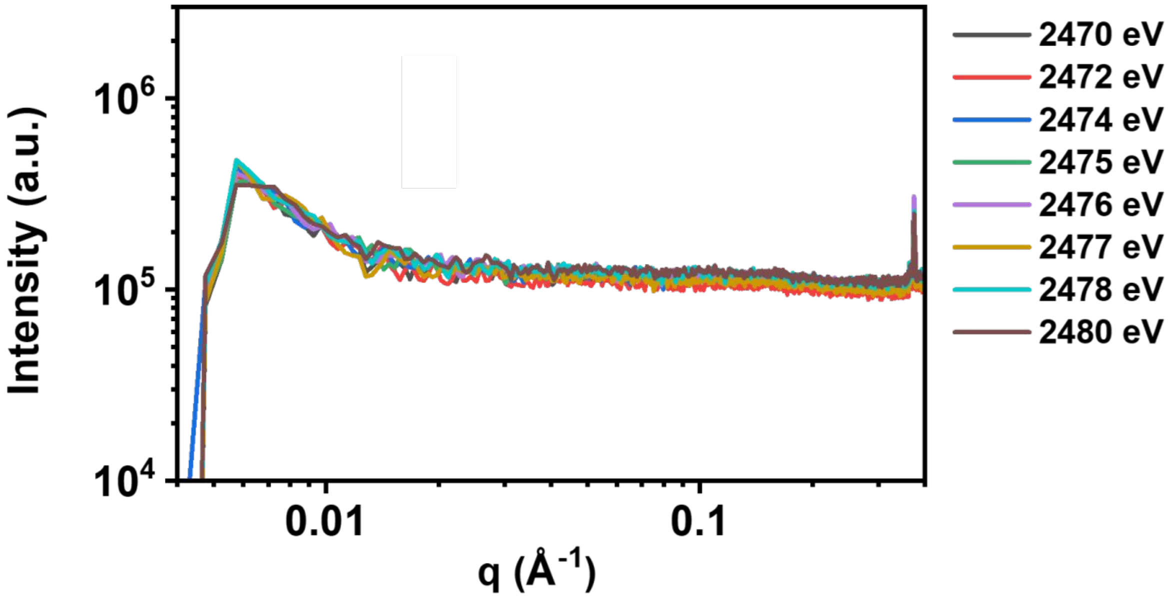

Figure S11. Tender X-ray scattering profile of solvent, TMB, at different energies (2470-2480 eV) near sulfur K-edge at 172 °C. The solvent didn't show E-dependent

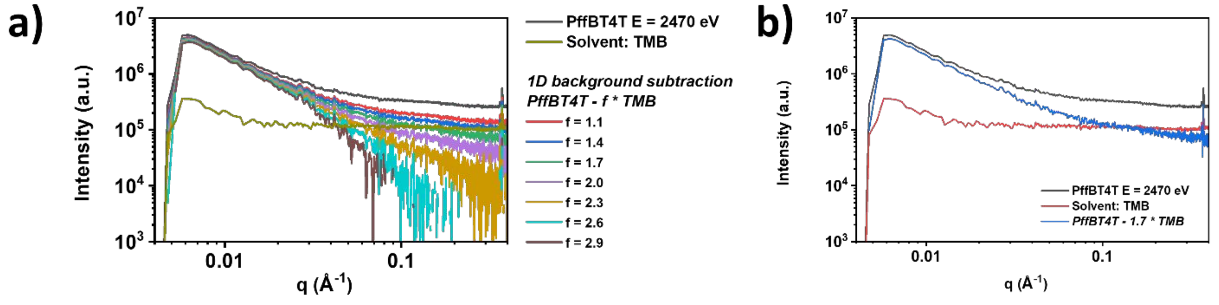

Figure S12. a) Tender X-ray scattering profile of PffBT4T solution and solvent, TMB, at 2470 eV and 172 °C, along with the PffBT4T profile after background subtraction from the solvent, TMB, scaled by factors from 1.1 to 2.9. b) Tender X-ray scattering profile of PffBT4T solution and solvent, TMB, at 2470 eV and 172 °C, along with the PffBT4T profile after selected background subtraction from the solvent, TMB, with factor of 1.7.

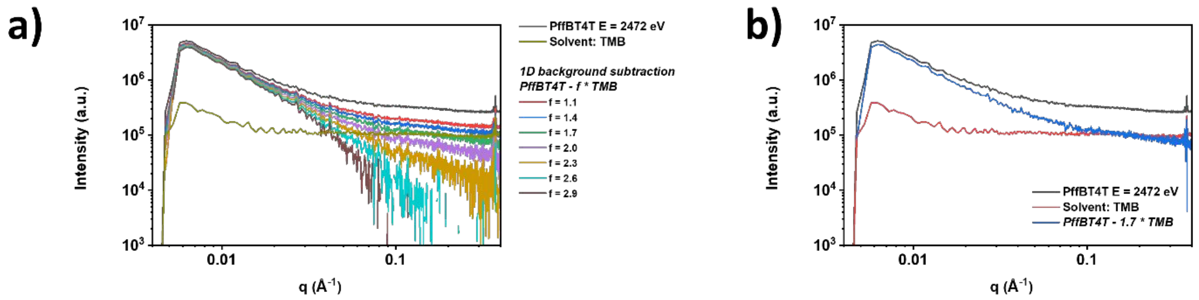

Figure S13. Tender X-ray scattering profile of PffBT4T solution and solvent, TMB, at 2472 eV and 172 °C, along with the PffBT4T profile after background subtraction from the solvent, TMB, scaled by factors from 1.1 to 2.9. b) Tender X-ray scattering profile of PffBT4T solution and solvent, TMB, at 2472 eV and 172 °C, along with the PffBT4T profile after selected background subtraction from the solvent, TMB, with factor of 1.7.

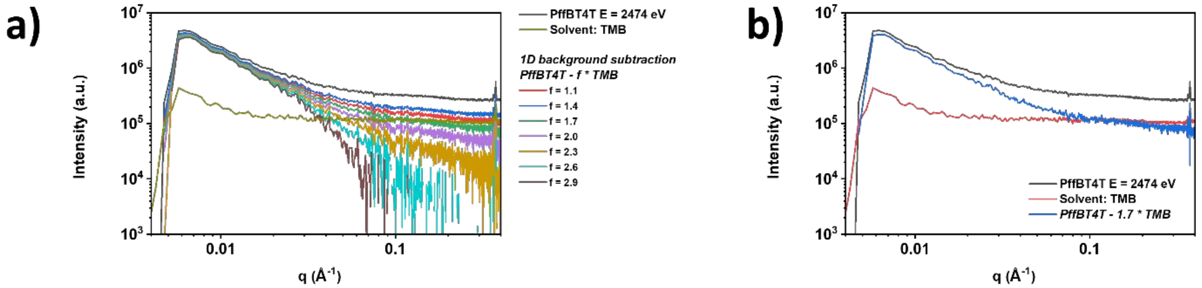

Figure S14. Tender X-ray scattering profile of PffBT4T solution and solvent, TMB, at 2474 eV and 172 °C, along with the PffBT4T profile after background subtraction from the solvent, TMB, scaled by factors from 1.1 to 2.9. b) Tender X-ray scattering profile of PffBT4T solution and solvent, TMB, at 2474 eV and 172 °C, along with the PffBT4T profile after selected background subtraction from the solvent, TMB, with factor of 1.7.

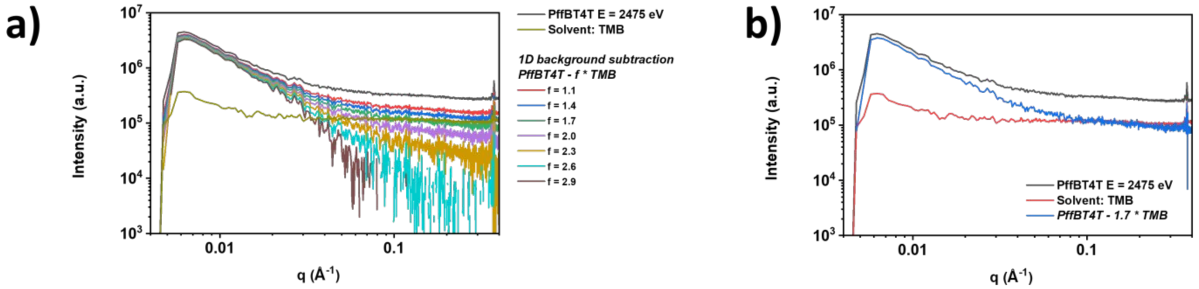

Figure S15. Tender X-ray scattering profile of PffBT4T solution and solvent, TMB, at 2475 eV and 172 °C, along with the PffBT4T profile after background subtraction from the solvent, TMB, scaled by factors from 1.1 to 2.9. b) Tender X-ray scattering profile of PffBT4T solution and solvent, TMB, at 2475 eV and 172 °C, along with the PffBT4T profile after selected background subtraction from the solvent, TMB, with factor of 1.7.

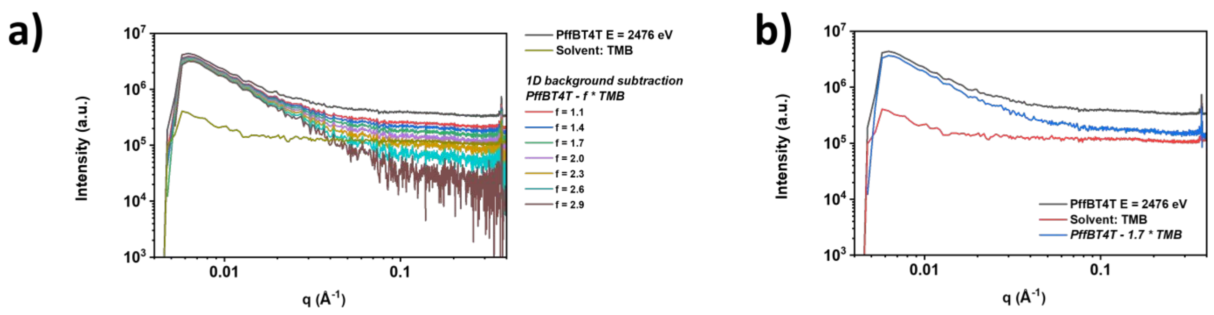

Figure S16. Tender X-ray scattering profile of PffBT4T solution and solvent, TMB, at 2476 eV and 172 °C, along with the PffBT4T profile after background subtraction from the solvent, TMB, scaled by factors from 1.1 to 2.9. b) Tender X-ray scattering profile of PffBT4T solution and solvent, TMB, at 2476 eV and 172 °C, along with the PffBT4T profile after selected background subtraction from the solvent, TMB, with factor of 1.7.

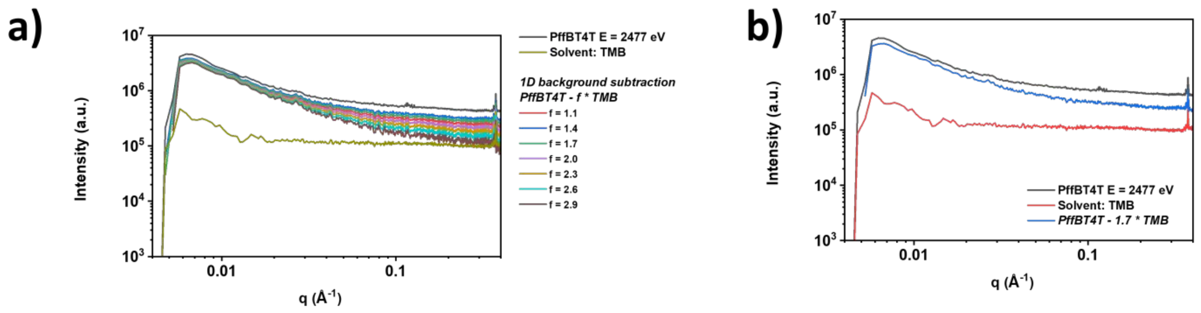

Figure S17. Tender X-ray scattering profile of PffBT4T solution and solvent, TMB, at 2477 eV and 172 °C, along with the PffBT4T profile after background subtraction from the solvent, TMB, scaled by factors from 1.1 to 2.9. b) Tender X-ray scattering profile of PffBT4T solution and solvent, TMB, at 2477 eV and 172 °C, along with the PffBT4T profile after selected background subtraction from the solvent, TMB, with factor of 1.7.

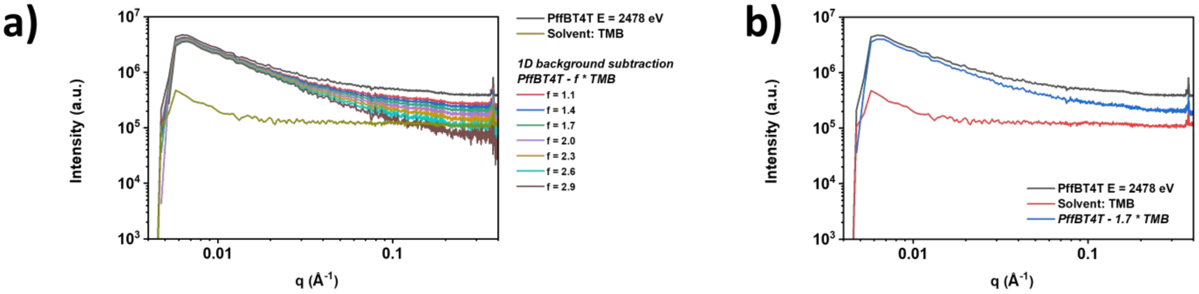

Figure S18. Tender X-ray scattering profile of PffBT4T solution and solvent, TMB, at 2478 eV and 172 °C, along with the PffBT4T profile after background subtraction from the solvent, TMB, scaled by factors from 1.1 to 2.9. b) Tender X-ray scattering profile of PffBT4T solution and solvent, TMB, at 2478 eV and 172 °C, along with the PffBT4T profile after selected background subtraction from the solvent, TMB, with factor of 1.7.

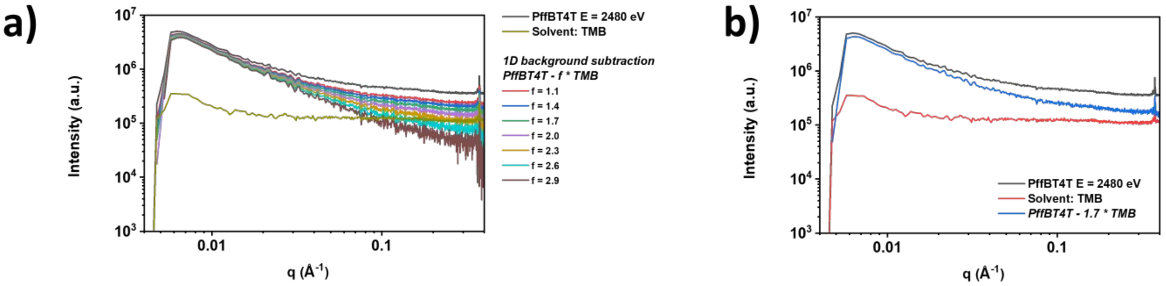

Figure S19. Tender X-ray scattering profile of PffBT4T solution and solvent, TMB, at 2480 eV and 172 °C, along with the PffBT4T profile after background subtraction from the solvent, TMB, scaled by factors from 1.1 to 2.9. b) Tender X-ray scattering profile of PffBT4T solution and solvent, TMB, at 2480 eV and 172 °C, along with the PffBT4T profile after selected background subtraction from the solvent, TMB, with factor of 1.7.

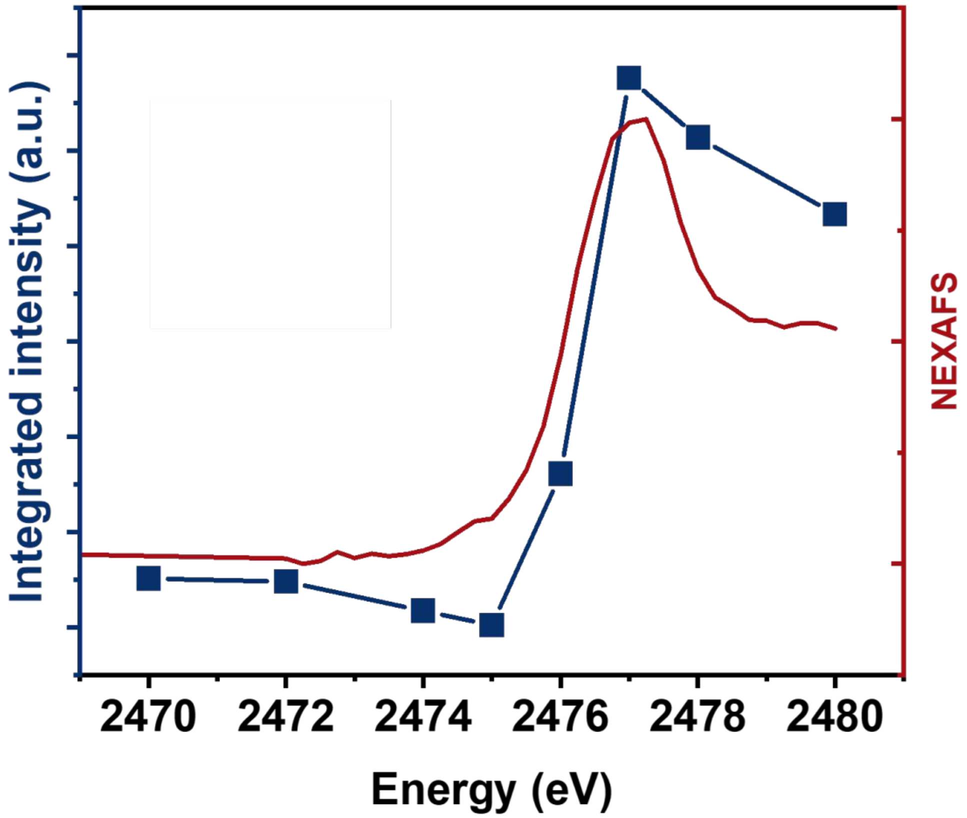

Figure S20. Integrated intensity of subtracted 1D scattering profile at different energies versus NEXAFS spectra result.

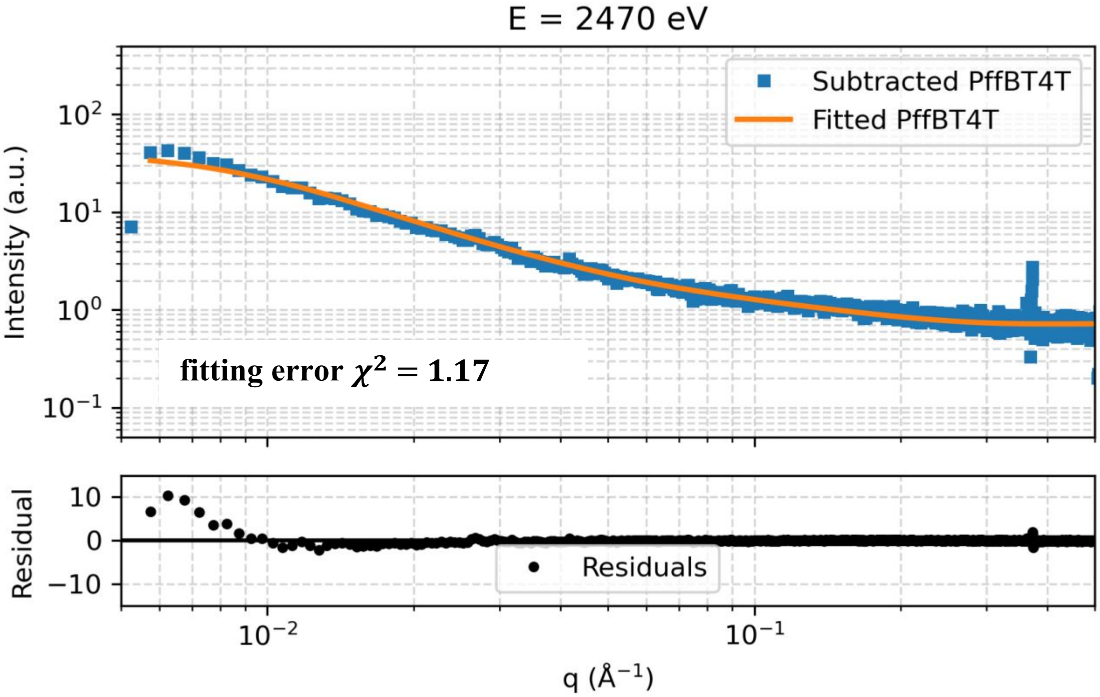

Figure S21. Scattering profiles (light blue scatter), best fits (dark blue line) and residual (black line) of the PffBT4T solution in trimethylbenzene (TMB) using the Flexible Cylinder Model at energies of 2470 eV and temperature of 172 °C (fitting error $\chi^2 = 1.17$).

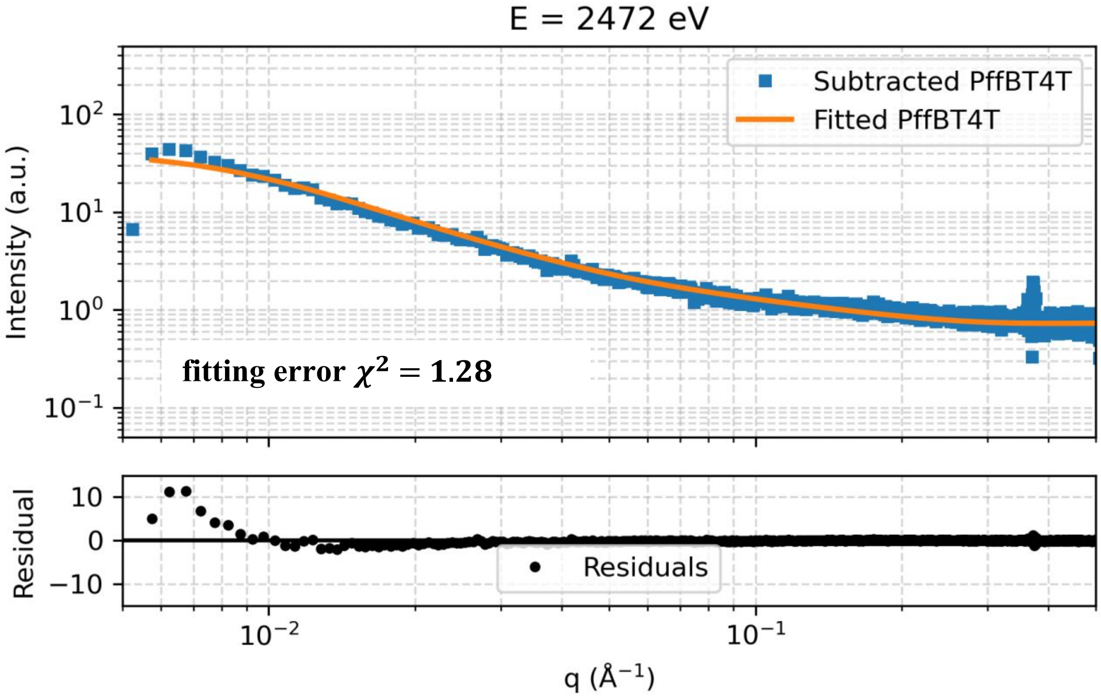

Figure S22. Scattering profiles (light blue scatter), best fits (dark blue line) and residual (black line) of the PffBT4T solution in trimethylbenzene (TMB) using the Flexible Cylinder Model at energies of 2472 eV and temperature of 172 °C. (fitting error $\chi^2 = 1.28$).

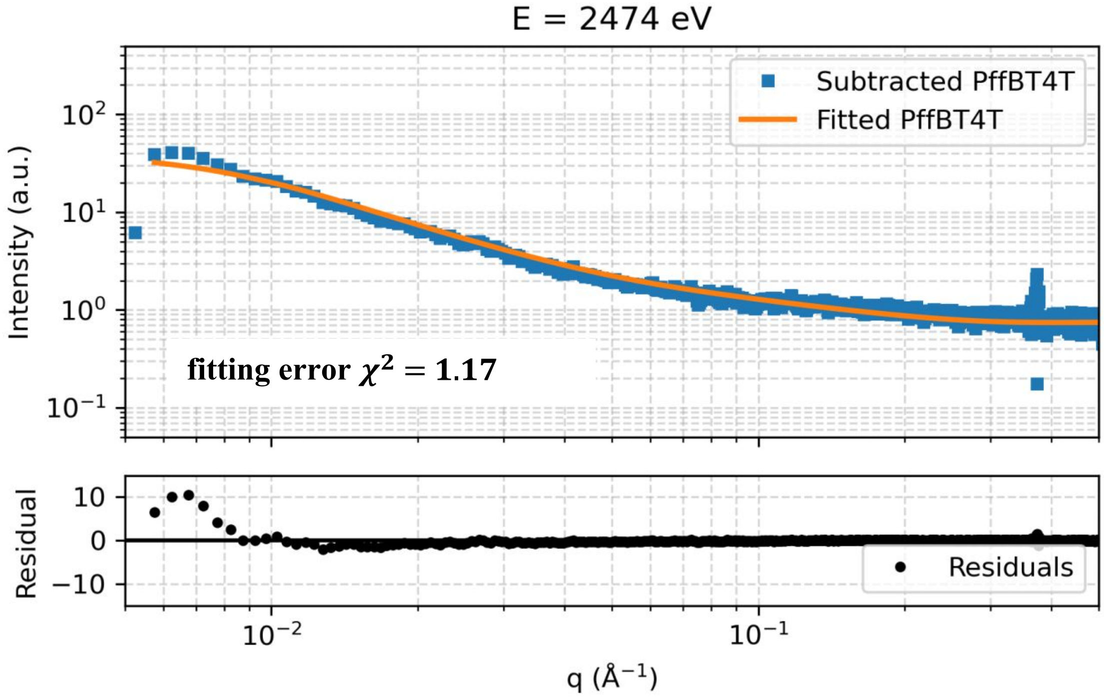

Figure S23. Scattering profiles (light blue scatter), best fits (dark blue line) and residual (black line) of the PffBT4T solution in trimethylbenzene (TMB) using the Flexible Cylinder Model at energies of 2474 eV and temperature of 172 °C. (fitting error $\chi^2 = 1.17$).

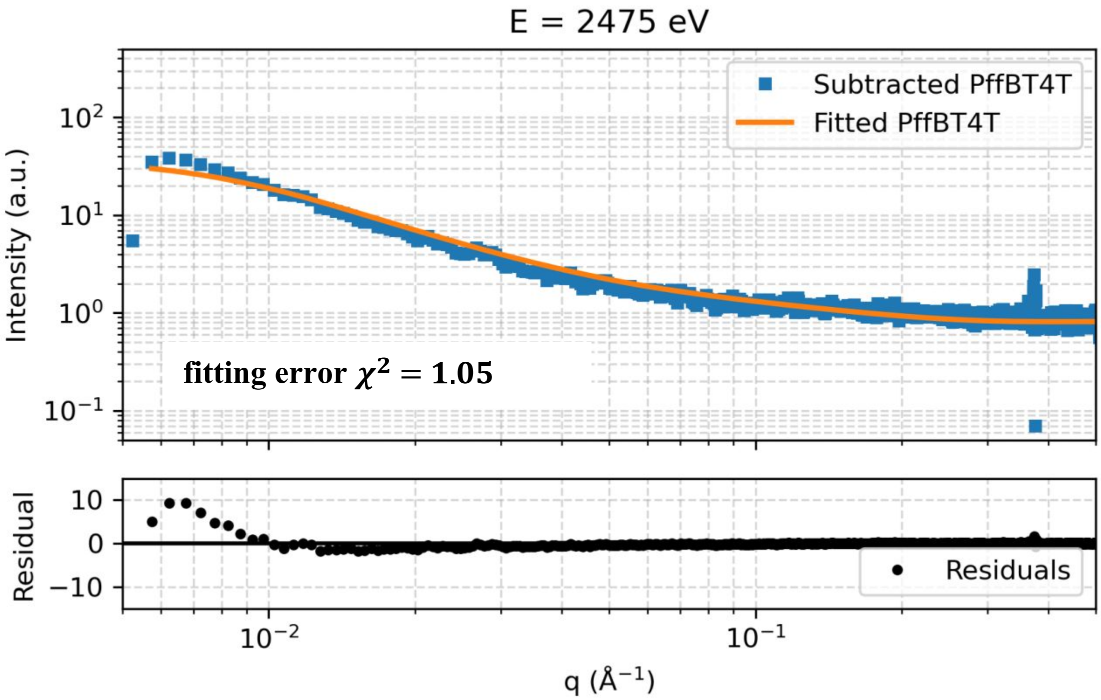

Figure S24. Scattering profiles (light blue scatter), best fits (dark blue line) and residual (black line) of the PffBT4T solution in trimethylbenzene (TMB) using the Flexible Cylinder Model at energies of 2475 eV and temperature of 172 °C. (fitting error $\chi^2 = 1.05$).

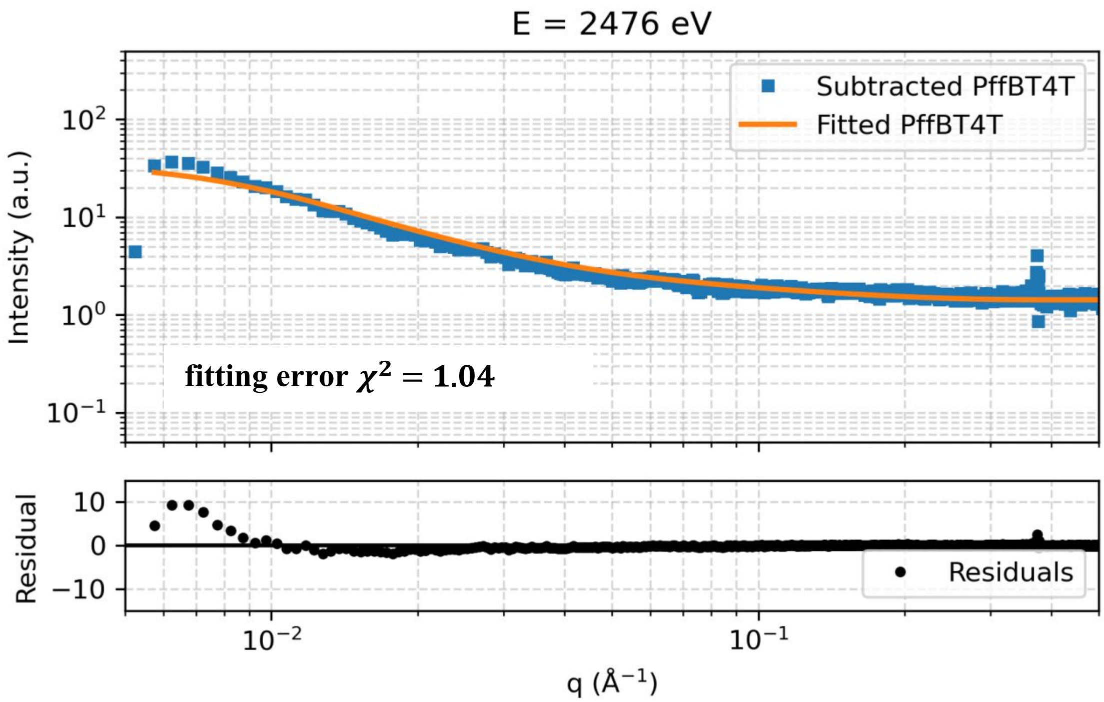

Figure S25. Scattering profiles (light blue scatter), best fits (dark blue line) and residual (black line) of the PffBT4T solution in trimethylbenzene (TMB) using the Flexible Cylinder Model at energies of 2476 eV and temperature of 172 °C. (fitting error $\chi^2 = 1.04$).

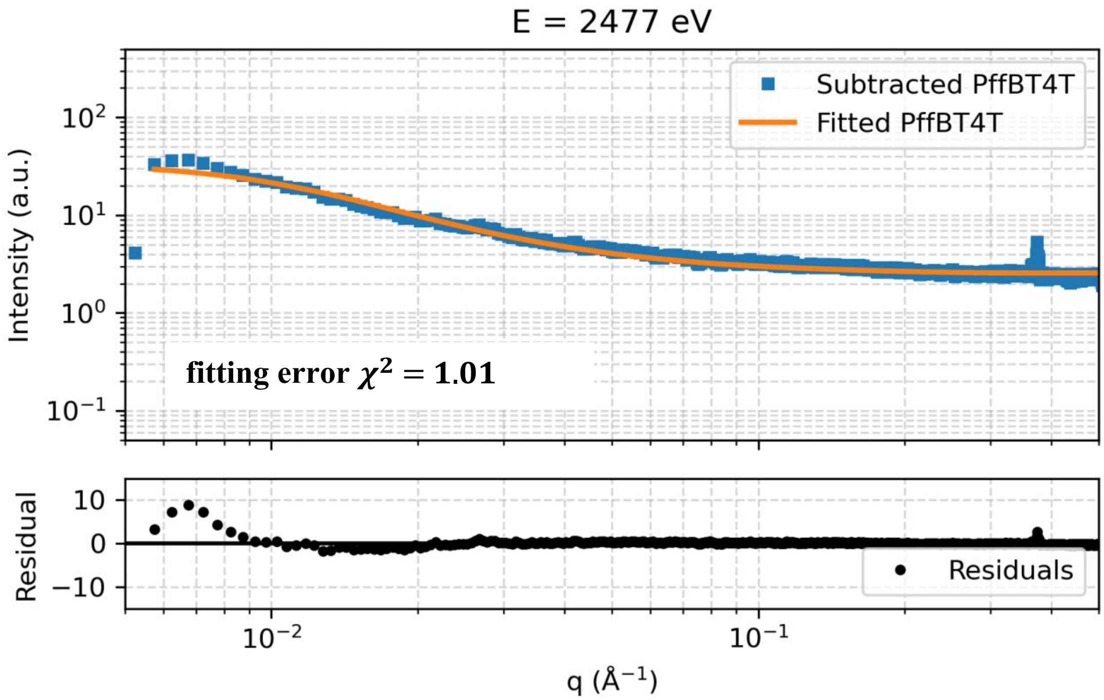

Figure S26. Scattering profiles (light blue scatter), best fits (dark blue line) and residual (black line) of the PffBT4T solution in trimethylbenzene (TMB) using the Flexible Cylinder Model at energies of 2477 eV and temperature of 172 °C. (fitting error $\chi^2 = 1.01$).

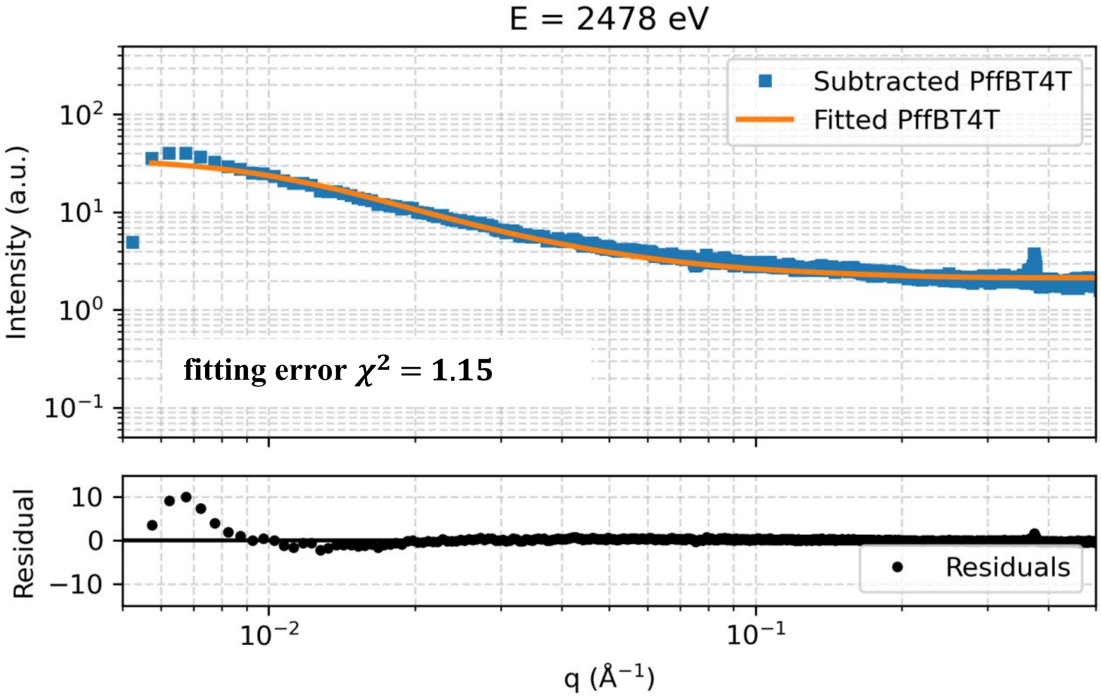

Figure S27. Scattering profiles (light blue scatter), best fits (dark blue line) and residual (black line) of the PffBT4T solution in trimethylbenzene (TMB) using the Flexible Cylinder Model at energies of 2478 eV and temperature of 172 °C. (fitting error $\chi^2 = 1.15$).

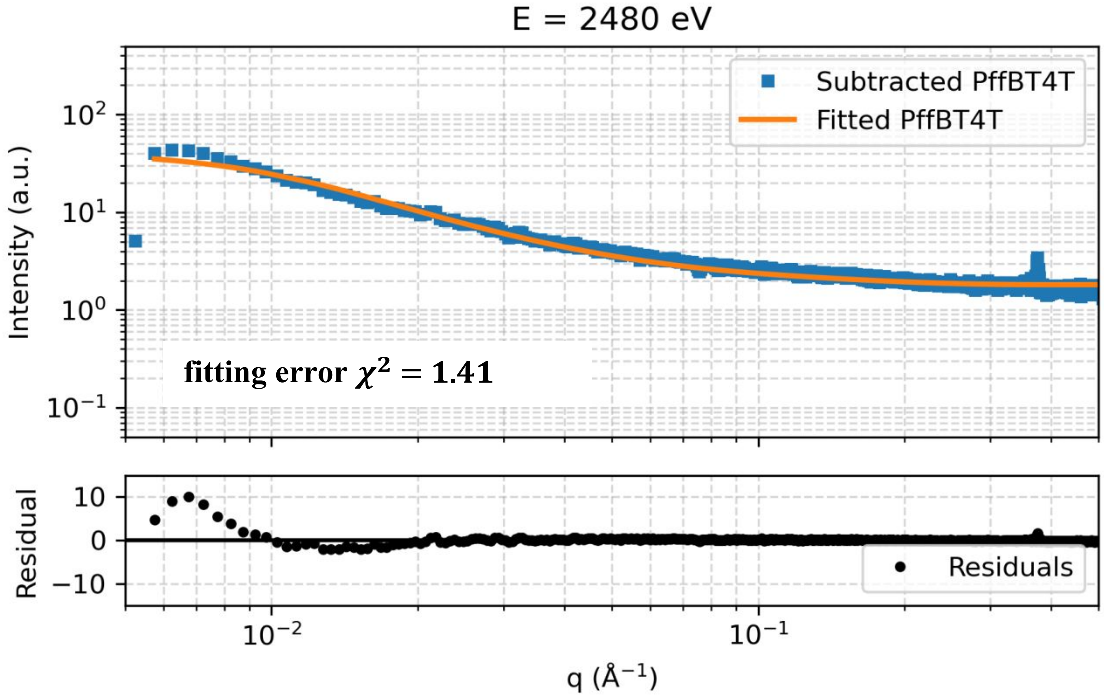

Figure S28. Scattering profiles (light blue scatter), best fits (dark blue line) and residual (black line) of the PffBT4T solution in trimethylbenzene (TMB) using the Flexible Cylinder Model at energies of 2480 eV and temperature of 172 °C. (fitting error $\chi^2 = 1.41$).

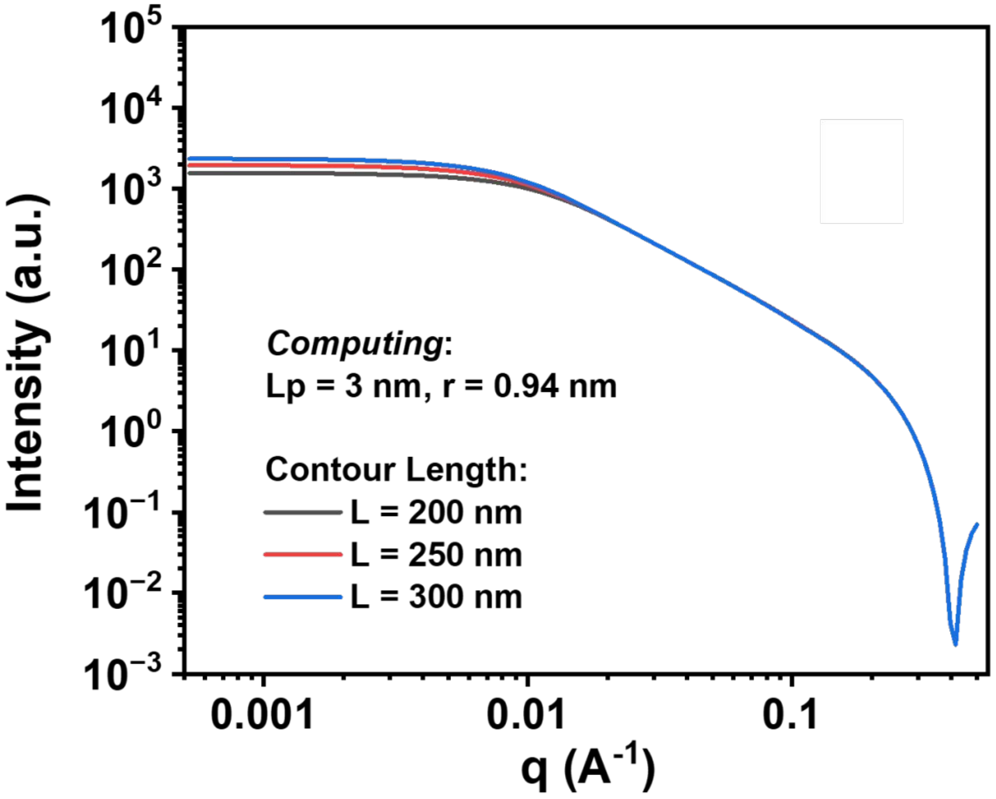

Figure S29. Simulated scattering curves using the flexible cylinder model with varying contour lengths (200, 250, and 300 nm), while keeping the persistence length (3 nm) and cylinder radius (0.94 nm) constant. The results show that changes in contour length affect only the low-q region, with negligible impact on the mid-q regime used to extract the persistence length (l□), supporting the robustness of l□ fitting against assumptions about contour length.

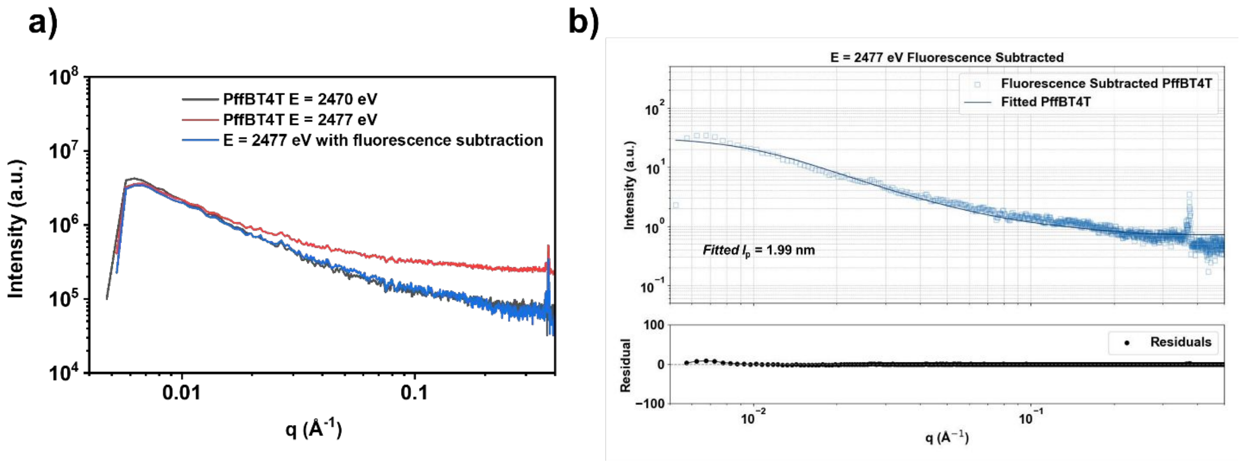

Figure S30. (a) Subtracted 1D tender X-ray scattering profile of PffBT4T at energy of 2477 eV (red) and after fluorescence correction (blue). Subtracted 1D tender X-ray scattering profile of PffBT4T at energy of 2470 eV (black). (b) Scattering profiles (light blue scatter), best fits (dark blue line) and residual (black line) of the PffBT4T solution after fluorescence correction in trimethylbenzene (TMB) using the Flexible Cylinder Model at energies of 2477 eV and temperature of 172 °C. *(fitting error* $\chi^2 = 1.01$*).*

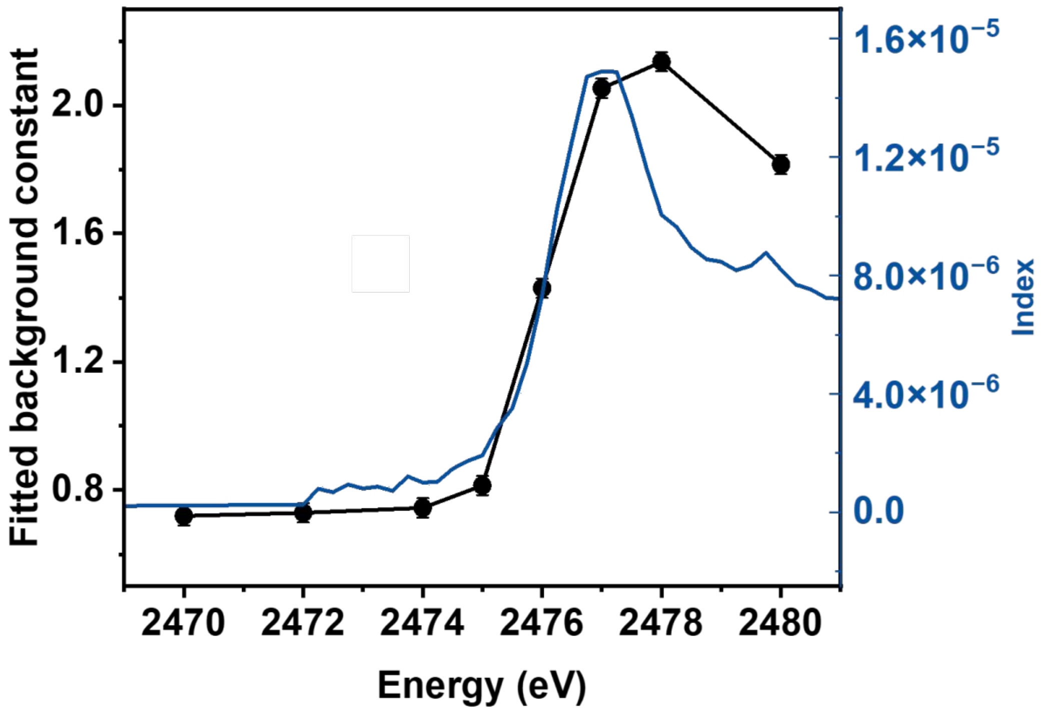

Figure S31. Fitted background constant versus beta (β) at different energies.
